# Effects of luseogliflozin and voglibose on high-risk lipid profiles and inflammatory markers in diabetes patients with heart failure

**DOI:** 10.1038/s41598-022-19371-6

**Published:** 2022-09-14

**Authors:** Kentaro Ejiri, Toru Miyoshi, Hajime Kihara, Yoshiki Hata, Toshihiko Nagano, Atsushi Takaishi, Hironobu Toda, Seiji Namba, Yoichi Nakamura, Satoshi Akagi, Satoru Sakuragi, Taro Minagawa, Yusuke Kawai, Nobuhiro Nishii, Soichiro Fuke, Masaki Yoshikawa, Kazufumi Nakamura, Hiroshi Ito, The MUSCAT-HF Study Investigators, Kentaro Ejiri, Toru Miyoshi, Kazufumi Nakamura, Hiroshi Ito, Hajime Kihara, Yoshiki Hata, Toshihiko Nagano, Atsushi Takaishi, Hironobu Toda, Seiji Namba, Yoichi Nakamura, Satoshi Akagi, Satoru Sakuragi, Taro Minagawa, Yusuke Kawai, Nobuhiro Nishii, Tetsuya Sato, Soichiro Fuke, Masaki Yoshikawa, Hiroyasu Sugiyama, Michio Imai, Naoki Gotoh, Tomonori Segawa, Toshiyuki Noda, Masatoshi Koshiji

**Affiliations:** 1 Department of Cardiovascular Medicine, Okayama University Graduate School of Medicine, Dentistry and Pharmaceutical Sciences, 2-5-1 Shikata-Cho, Kita-Ku, Okayama, 700-8558 Japan; 2 Department of Internal Medicine, Tamano City Hospital, Tamano, Japan; 3 Department of Internal Medicine, Kihara Cardiovascular Clinic, Asahikawa, Japan; 4 Department of Cardiology, Minamino Cardiovascular Hospital, Hachioji, Japan; 5 Department of Internal Medicine, Iwasa Hospital, Gifu, Japan; 6 Department of Cardiology, Mitoyo General Hospital, Kanonji, Japan; 7 Department of Internal Medicine, Okayama East Neurosurgery Hospital, Okayama, Japan; 8 Department of Cardiology, Okayama Rosai Hospital, Okayama, Japan; 9 Department of Cardiovascular Medicine, Specified Clinic of Soyokaze Cardiovascular Medicine and Diabetes Care, Matsuyama, Japan; 10 Department of Internal Medicine, Akaiwa Medical Association Hospital, Akaiwa, Japan; 11 Department of Cardiovascular Medicine, Iwakuni Clinical Center, Iwakuni, Japan; 12 Department of Internal Medicine, Minagawa Cardiovascular Clinic, Gifu, Japan; 13 Department of Cardiovascular Medicine, Okayama City Hospital, Okayama, Japan; 14 Department of Internal Medicine, Yoshinaga Hospital, Bizen, Japan; 15 Department of Cardiovascular Medicine, Japanese Red Cross Okayama Hospital, Okayama, Japan; 16 Department of Cardiology, Fukuyama City Hospital, Fukuyama, Japan; 17 Okayama University Graduate School of Medicine, Dentistry and Pharmaceutical Sciences, Okayama, Japan; 18 Tamano City Hospital, Tamano, Japan; 19 Kihara Cardiovascular Clinic, Asahikawa, Japan; 20 Minamino Cardiovascular Hospital, Hachioji, Japan; 21 Iwasa Hospital, Gifu, Japan; 22 Mitoyo General Hospital, Kanonji, Japan; 23 Okayama East Neurosurgery Hospital, Okayama, Japan; 24 Okayama Rosai Hospital, Okayama, Japan; 25 Specified Clinic of Soyokaze Cardiovascular Medicine and Diabetes Care, Matsuyama, Japan; 26 Akaiwa Medical Association Hospital, Akaiwa, Japan; 27 Iwakuni Clinical Center, Iwakuni, Japan; 28 Minagawa Cardiovascular Clinic, Gifu, Japan; 29 Okayama City Hospital, Okayama, Japan; 30 Yoshinaga Hospital, Bizen, Japan; 31 Japanese Red Cross Okayama Hospital, Okayama, Japan; 32 Fukuyama City Hospital, Fukuyama, Japan; 33 Imai Heart Clinic, Ibaraki, Japan; 34 Gotoh Clinic, Gifu, Japan; 35 Asahi University Hospital, Gifu, Japan; 36 Gifu Prefectural General Medical Center, Gifu, Japan; 37 Gifu Seiryu Hospital, Gifu, Japan

**Keywords:** Cardiology, Cardiovascular biology, Cardiovascular diseases, Dyslipidaemias

## Abstract

Sodium-glucose cotransporter 2 inhibitors could reduce cardiovascular events in patients with heart failure irrespective of diabetes status. In this prespecified sub-analysis of randomised-controlled trial, we investigated the efficacy of luseogliflozin (2.5 mg daily), a sodium–glucose cotransporter 2 inhibitor, with that of voglibose (0.6 mg daily), an alpha-glucosidase inhibitor, on high-risk lipid profile and inflammatory markers in patients with type-2 diabetes and heart failure. Among the 157 patients studied, there were no significant differences in the mean malondialdehyde LDL or small-dense LDL cholesterol levels between the luseogliflozin and voglibose groups (percent change: 0.2% vs. − 0.6%, p = 0.93; − 1.7% vs. − 8.6%, p = 0.21) after 12 weeks in comparison to levels at the baseline. No significant difference was observed between the two groups in the adiponectin and high-sensitivity C-reactive protein levels after 12 weeks compared to the baseline levels (percent change, − 1.6% vs. − 4.0% and 22.5% vs. 10.0%; p = 0.52 and p = 0.55, respectively). In conclusion, in patients with type-2 diabetes and heart failure, compared to voglibose, luseogliflozin did not significantly improve the high-risk lipoprotein profile including malondialdehyde LDL and small-dense LDL cholesterol or the levels of inflammatory markers, including adiponectin and high-sensitivity C-reactive protein.

**Trial registration:** Trial number: UMIN-CTR, UMIN000018395; Registered 23 July 2015; URL: https://www.umin.ac.jp/ctr/index.htm.

## Introduction

Sodium–glucose cotransporter-2 (SGLT2) inhibitors are therapeutic agents against diabetes mellitus that lower serum glucose levels by promoting urinary glucose excretion. Because SGLT2 inhibitors have shown efficacy in preventing hospitalisation for heart failure in patients with or without diabetes in clinical trials^1–6^, their indication has been expanded from diabetes to heart failure. Beyond the initial hypoglycaemic effect elicited by SGLT2 inhibitors^7^, they also exert other effects, such as haematopoietic^8^, diuretic^9^, antihypertensive, and sympathetic nerve activity-inhibitory effects^10^. Although various hypotheses have been proposed to explain the mechanisms underlying the lowering of the risk for cardiovascular events by SGLT2 inhibitors, their exact mechanisms remains uncertain. Several clinical studies investigating the efficacy of SGLT2 inhibitors^1–6,11,12^ have reported consistent positive effects of SGLT2 inhibitors, such as lowering serum glucose, preventing hospitalisation for heart failure, and renal protection. Conversely, inconsistent results were observed regarding the effects of SGLT2 inhibitors in preventing atherosclerotic cardiovascular disease (ASCVD), including cardiac death, myocardial infarction, and stroke^13^. Dyslipidaemia is a potential risk factor for ASCVD, and previous reports suggest that SGLT2 inhibitors might increase low-density lipoprotein (LDL) cholesterol levels^14^. Therefore, the effect of SGLT2 inhibitors on lipid profiles may be less effective in lowering the risk for ASCVD than that for heart failure or renal protection. However, only a few studies have investigated the effects of SGLT2 inhibitors on lipid profiles in patients with cardiac disease. One small sample-sized study reported that dapagliflozin suppressed small-dense LDL levels and increased LDL cholesterol compared to sitagliptin, dipeptidyl-peptidase IV inhibitor in patients with type-2 diabetes^15^. Furthermore, whether SGLT2 inhibitors have an anti-inflammatory effect is also unknown. This study aimed to compare the effects of an SGLT2 inhibitor with an alpha-glucosidase inhibitor on atherogenic risk factors, including high-risk lipid profile and inflammatory markers related to the incidence of ASCVD in diabetes patients with heart failure.

## Methods

### Study design

This study was designed as a prespecified sub-analysis of a randomised-controlled trial (RCT; the Management of Diabetic Patients with Chronic Heart Failure and Preserved Left Ventricular Ejection Fraction: MUSCAT-HF trial). Details of the study design have been published previously^16,17^ (Additional file 1). The MUSCAT-HF trial was a multicentre, prospective, open-label, RCT for comparing the effect of luseogliflozin (2.5 mg once daily) with that of voglibose (0.2 mg three times daily) on left ventricular loads in patients with type 2 diabetes and heart failure with preserved ejection fraction. The study was approved by the Okayama University Graduate School of Medicine, Dentistry and Pharmaceutical Sciences; the Okayama University Hospital Ethics Committee; the Tamano City Hospital Ethics Committee; the Okayama University Hospital Ethics Committee; the Mitoyo General Hospital Ethics Committee; the Okayama Rosai Hospital Ethics Committee; the Iwakuni Clinical Center Ethics Committee; the Okayama City Hospital Ethics Committee; the Japanese Red Cross Okayama Hospital Ethics Committee; and the Fukuyama City Hospital Ethics Committee. The study was also approved by the Okayama University Hospital Ethics Committee in other participating centres which did not organize own ethics committee. The investigation conforms to the principles outlined in the Declaration of Helsinki. This trial was registered in the University Hospital Medical Information Network Clinical Trial Registry on 23/07/2015 (UMIN-CTR, UMIN000018395).

Members of the Steering Committee also designed the study and are responsible for its execution (details are listed in the Additional file 1). Significant adverse events that occurred within 30 days after the final administration of the study drug or after 30 days with suspicion of association with the study drug, as well as all pregnancies, were immediately reported to the Steering Committee and the sponsor by the investigators, in accordance with the guidelines for good clinical practice.

### Participants

Patients aged 20 years and older requiring additional treatment for type 2 diabetes (despite ongoing treatment) and heart failure with preserved ejection fraction were eligible for participation in the study. Heart failure with preserved ejection fraction was defined as a left ventricular ejection fraction ≥ 45%, a b-type natriuretic peptide (BNP) concentration ≥ 35 pg/ml, and any related symptoms, such as shortness of breath, orthopnoea, and leg oedema. The criterion for BNP concentration was based on the definition of chronic heart failure by the European Society of Cardiology guidelines, which includes BNP concentrations ≥ 35 pg/ml^18^. The exclusion criteria were as follows: patients with BNP concentrations < 35 pg/ml; undergoing treatment with alpha-glucosidase inhibitors, SGLT2 inhibitors, glinides, or high-dose sulfonylurea; having renal insufficiency (estimated glomerular filtration rate [eGFR] < 30 ml/min/1.73 m^2^); a history of severe ketoacidosis or diabetic coma within 6 months before participation; poorly controlled type-2 diabetes (haemoglobin A1c [HbA1c] > 9.0%); and hypertension (see full exclusion criteria in Data S1). All participants provided written informed consent prior to participation. The study candidates were assessed for eligibility within 4 weeks before enrolment.

In this prespecified subanalysis, the effect of luseogliflozin or voglibose on the atherogenic lipid profile and inflammatory markers was evaluated in all patients who were administered the study drugs. Additionally, subgroup analyses were performed in patients with prior ASCVD, dyslipidaemia, or statin therapy at baseline.

### Interventions and study procedures

Patients fulfilling all the criteria and who provided written informed consent to participate in this study were enrolled and subsequently randomised (1:1) to receive luseogliflozin (2.5 mg once daily) or voglibose (0.2 mg three times daily), in addition to their background medication. Luseogliflozin is an SGLT2 inhibitor, which has 1600-fold selectivity for SGLT2 over SGLT1^19^, and is currently approved or marketed in Japan, but not in North America or European countries. Randomisation was performed using a computer-generated random sequence web-response system. The patients were stratified by age (< 65 years, ≥ 65 years), baseline haemoglobin A1c (HbA1c) values (< 8.0%, ≥ 8.0%), baseline BNP concentrations (< 100 pg/ml, ≥ 100 pg/ml), baseline renal function (eGFR ≥ 60 ml/min/1.73 m^2^, < 60 ml/min/1.73 m^2^), use of thiazolidine (yes or no), and presence or absence of atrial fibrillation and flutter at screening.

Laboratory data were evaluated at 12 weeks after initiating the treatment, while safety and tolerability were assessed at 4 and 12 weeks after treatment initiation by interview, physical examination, and general laboratory tests. After 12 weeks, follow-up treatment was continued for an additional 12 weeks in patients who agreed. If a patient’s glycaemic control worsened after 4 weeks, the investigator increased the dose of the allocated treatment (5 mg luseogliflozin once daily or 0.3 mg voglibose three times daily) and other specific anti-diabetes drugs, except for sulfonylureas. The investigators were also encouraged to treat all other cardiovascular risk factors according to the local standard of care. Under the following circumstances, the investigators evaluated the data and patients’ vital signs: (1) discontinuation of study treatment; (2) dose increase of specific treatment for heart failure; (3) initiation of a new treatment for heart failure; and (4) withdrawal from the study. The permitted medications for treating heart failure included angiotensin-converting enzyme inhibitors, angiotensin-receptor blockers, beta-blockers, diuretics, and mineralocorticoid/aldosterone receptor antagonists.

### Outcomes

The definitions of the major outcomes in the MUSCAT-HF trial have been published elsewhere^16,17^. The primary outcome of this study was the difference from baseline in atherogenic lipoproteins including the levels of malondialdehyde low-density lipoprotein (MDA-LDL), small-dense LDL cholesterol, inflammatory markers (adiponectin and high-sensitivity C-reactive protein [CRP]) after 12 weeks of treatment between the two drugs. Secondary outcomes were the change from baseline in the lipid profile including total cholesterol, high-density lipoprotein (HDL) cholesterol, LDL cholesterol, and triglyceride level after treatment. LDL cholesterol was calculated using the Friedewald equation^20^.

Blood samples were sent to an external laboratory (SRL, Tokyo, Japan) for analysis. The levels of total cholesterol, triglyceride, HDL cholesterol, LDL cholesterol, haemoglobin, and high-sensitivity CRP were assayed using standard laboratory procedures. MDA-LDL levels were assessed by enzyme-linked immunosorbent assay (Sekisui Medical Co., Tokyo, Japan)^21^. For MDA-LDL measurement, inter- and intra-assay coefficients of variation were 6.5% and 9.0%, respectively^22^. A homogeneous assay was used for the direct measurement of small-dense LDL cholesterol levels (sd-LDL-EX “Seiken”, Denka Seiken, Tokyo, Japan)^23^. The intra- and inter-assay coefficients of variation for the small-dense LDL cholesterol assay were 1.3% and 3.1%, respectively. The serum concentration of adiponectin was evaluated using a latex particle-enhanced turbidimetric immunoassay on an automated analyser (Adiponectin Latex Kit, Otsuka Pharmaceutical Co., Ltd., Tokyo, Japan)^24^. The within- and between-run coefficients of variation were 0.6% and 1.3%, respectively.

### Statistical analysis

Data were analysed according to a predefined statistical analysis plan, and an independent statistician verified and replicated the analyses. Continuous variables are presented as the mean ± standard deviation or as median with the interquartile range, depending on the Shapiro–Wilks test for normality. Categorical variables are presented as absolute values and proportions (%).

Analysis of variance or the Kruskal–Wallis test was used to compare continuous variables among the study groups. The χ^2^ test was used to compare categorical variables among the groups. For the primary outcome analysis, an analysis of covariance (ANCOVA) was performed to assess the changes in the ratios of MDA-LDL, small-dense LDL cholesterol, adiponectin, and high-sensitivity CRP concentrations after 12 weeks from the baseline. Adjusted covariates included the assigned treatment (luseogliflozin or voglibose), baseline age (< 65 or ≥ 65 years), baseline HbA1c values (< 8.0 or ≥ 8.0%), baseline BNP concentration (< 100 or ≥ 100 pg/ml), baseline renal function (eGFR ≥ 60 or < 60 ml/min/1.73 m^2^), use of thiazolidine at baseline, and the presence or absence of atrial fibrillation and atrial flutter at baseline as the stratified factors of randomisation. A similar method was used to analyse the secondary outcomes (total cholesterol, HDL cholesterol, LDL cholesterol, and triglyceride). To assess time-dependent changes in each biomarker (compared to baseline) after treatment within the drug group, mixed-effect linear regression models with compound symmetry correlation matrix were used to account for the within-participant correlation, thus adjusting for the baseline value of the biomarker in addition to the same covariates as in the ANCOVA model. Furthermore, the same analyses were conducted for subgroups stratified by relevant baseline characteristics, including prior ASCVD, dyslipidaemia, and statin use at baseline. All comparisons and analyses were two-sided with *p* values < 0.05 considered to reflect statistically significant differences. All statistical analyses were performed using IBM SPSS Statistics 24 (IBM, Armonk, NY), Stata/SE 15.1 for the Mac (StataCorp, College Station, TX), and R 4.1.2 (The R Foundation for Statistical Computing, Vienna, Austria).

### Ethics approval and consent to participate

The study was approved by the Okayama University Graduate School of Medicine, Dentistry and Pharmaceutical Science, the Okayama University Hospital Ethics Committee, and the ethics committee of each participating centre. The investigation conforms with the principles outlined in the Declaration of Helsinki. This trial was registered in the University Hospital Medical Information Network Clinical Trial Registry on 23/07/2015 (UMIN-CTR, UMIN000018395).

## Results

### Patient background

**Figure 1:**
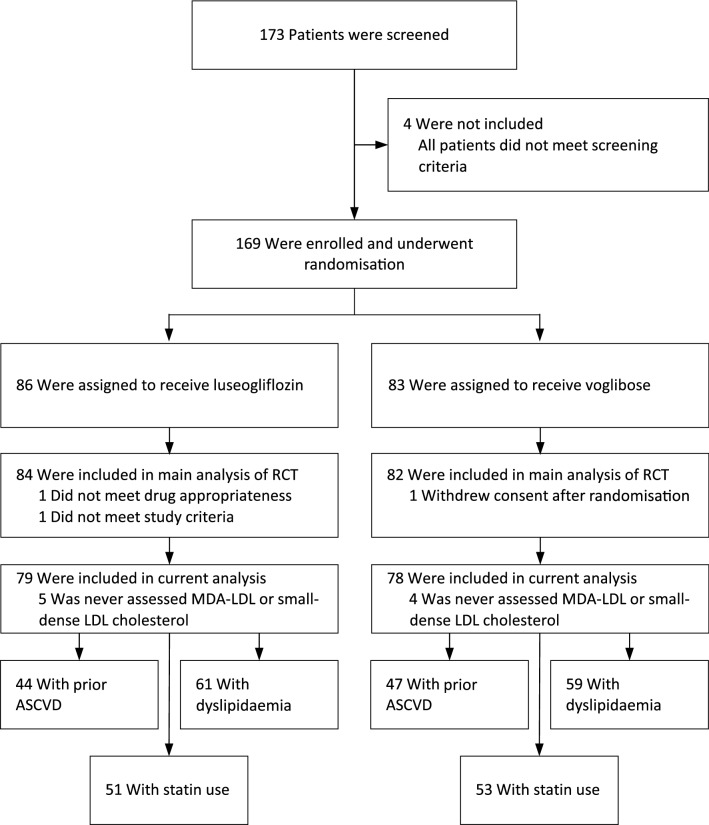
Study flow diagram. *RCT* randomised-controlled trial, *MDA-LDL* malondialdehyde low-density lipoprotein, *ASCVD* atherosclerotic cardiovascular disease.

Between December 2015 and September 2018, we screened 173 patients from 16 hospitals and clinics for participation in this study. A total of 169 patients were enrolled in this study. Of these patients, 86 were assigned to receive luseogliflozin and 83 were assigned to receive voglibose. Three patients (1.8%) who did not receive any doses of these drugs were prospectively excluded from all analyses. Nine patients (5.3%) who were not evaluated for MDA-LDL or small-dense LDL cholesterol levels during the study period were also excluded. The remaining 157 patients (79 in the luseogliflozin group and 78 in the voglibose group) for whom laboratory data measurements were assessed at least once, were included in this sub-analysis (Fig. 1). High adherence to drugs was observed during each hospital visit among the study population; the mean adherence rate was 96.8% (luseogliflozin: 98.3%, voglibose: 95.2%). During the 12-week visit, the drug dose was changed for 10 patients (13%) in the voglibose group due to clinical reasons, while no changes were made in the luseogliflozin group.

The baseline characteristics of the patients are shown in Table 1. The baseline variables were similar between the luseogliflozin and voglibose groups, except for the patients’ age and LDL cholesterol level. The mean age was significantly lower in patients in the luseogliflozin group than in the voglibose group (p = 0.017). The proportions of patients with prior atherosclerotic cardiovascular disease were 59% and 62%, the proportions of those with dyslipidaemia were 80% and 75%, and the proportions of those with statin therapy were 65% and 67% in the luseogliflozin and voglibose groups, respectively. There was no significant difference in baseline medications (including fibrate, ezetimibe, and any diabetic agent) between the two groups. The mean HbA1c concentration did not differ significantly between the two groups. At baseline, the mean MDA-LDL was 96.3 vs. 97.4 U/l and the mean small-dense LDL cholesterol was 32.6 vs. 32.5 IU/l between the luseogliflozin and voglibose groups, respectively. No significant differences were observed in any atherogenic or inflammatory marker levels between the two groups, whereas the mean of LDL cholesterol at baseline was significantly lower in the luseogliflozin group than that in the voglibose group (86.4 vs. 97.5 mg/dl, p = 0.03).

**Table 1:** Baseline patient characteristics.

Variables	Luseogliflozin group (n = 79)	Voglibose group (n = 78)	p value
**Age, y**	71.8 ± 7.8	74.9 ± 7.6	0.01
Median (interquartile range)	72 (68–78)	75 (70–79)	0.02
> 60 y, n (%)	73 (92.4)	76 (97.4)	0.15
Male, n (%)	54 (68.4)	45 (57.7)	0.17
Body weight, kg	65.0 ± 12.7	63.1 ± 12.4	0.35
Body mass index, kg/m^2^	25.4 ± 4.4	25.1 ± 4.2	0.72
Waist circumflex, cm	92.5 ± 11.5	91.1 ± 11.9	0.47
**NYHA class, n (%)**			0.43
I	0	0	
II	77 (96.2)	77 (98.7)	
III	3 (3.8)	1 (1.3)	
IV	0	0	
Duration of diabetes, months	72 (24–130)	72 (36–138)	0.85
**Prior diagnoses, n (%)**			
Hypertension	70 (90.9)	61 (79.2)	0.04
Hyperuricemia	18 (23.4)	23 (29.9)	0.36
Cardiovascular disease	44 (57.1)	47 (51.6)	0.62
Dyslipidaemia	61 (79.2)	59 (79.6)	0.70
Chronic kidney disease	27 (35.1)	27 (35.1)	1.0
Hepatic disorder	8 (10.4)	3 (3.9)	0.12
Atrial fibrillation or flutter	17 (21.5)	14 (17.9)	0.57
**Medications, n (%)**			
Statin	51 (64.6)	53 (67.9)	0.65
Fibrate	2 (2.5)	5 (6.4)	0.28
Ezetimibe	15 (19)	13 (16.7)	0.70
ACE inhibitor or ARB	50 (63.3)	45 (57.7)	0.47
Beta-blocker	49 (62.0)	44 (47.3)	0.47
Anti-diabetic medication	51 (65.4)	48 (61.5)	0.62
**Hemodynamic parameters**			
Systolic blood pressure, mmHg	132.4 ± 17.3	128.3 ± 14.4	0.11
Diastolic blood pressure, mmHg	72.4 ± 11.2	70.9 ± 10.4	0.40
Heart rate, bpm	68.8 ± 12.3	70.7 ± 11.2	0.36
**Laboratory data**			
Haemoglobin A1c, %	7.0 ± 0.7	6.9 ± 0.8	0.35
Haemoglobin, g/dl	13.6 ± 1.7	13.1 ± 1.5	0.057
Haematocrit, %	41.5 ± 4.8	40.2 ± 4.2	0.09
Blood urea nitrogen, mg/dl	17.4 ± 5.4	19.3 ± 6.0	0.046
Serum creatinine, mg/dl	0.94 ± 0.30	0.96 ± 0.30	0.63
Estimated GFR, ml/min/1.73 m^2^	60.9 ± 19.6	56.2 ± 16.2	0.11
BNP, pg/ml	62.8 (45.8–110.0)	75.5 (42.4–119.5)	0.86
**Atherogenic lipid and inflammatory profile**			
MDA-LDL, U/l	95.6 (34.7)	97.0 (38.5)	0.81
Small-dense LDL cholesterol, mg/dl	32.4 (14.1)	32.5 (15.9)	0.98
Adiponectin, µg/ml	8.9 (7.1–12.8)	10.1 (7.4–17.6)	0.08
High-sensitivity CRP, mg/l	0.91 (0.41–1.79)	0.73 (0.25–1.66)	0.71
Total cholesterol, mg/dl	175.6 (32.5)	183.4 (39.1)	0.18
HDL cholesterol, mg/dl	54.8 (15.7)	56.3 (17.3)	0.57
LDL cholesterol, mg/dl	86.4 (28.4)	97.5 (32.4)	0.03
Triglyceride, mg/dl	138.0 (99.0–221.0)	128.0 (88.5–178.0)	0.19

Data are presented as the mean ± standard deviation, n (%), or median (interquartile range).

*ACE* angiotensin-converting enzyme, *ARB* angiotensin-receptor blocker, *BNP* b-type natriuretic peptide, *CRP* C-reactive protein, *GFR* glomerular filtration rate, *HDL* high-density lipoprotein, *MDA-LDL* malondialdehyde low-density lipoprotein, *MRA* mineralocorticoid receptor antagonist, *NYHA* New York Heart Association.

### Primary outcome

**Figure 2:**
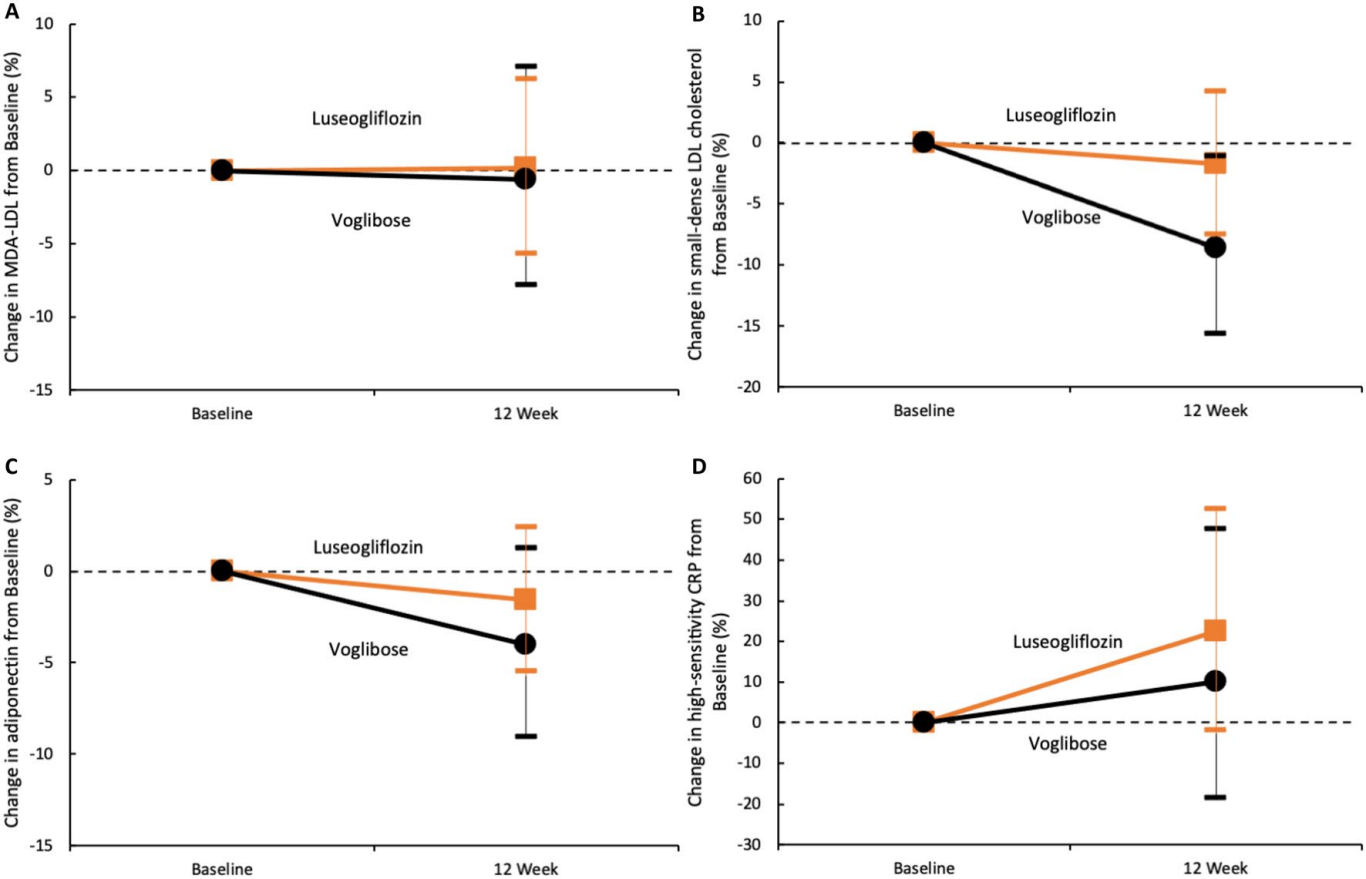
Changes in the high-risk lipid profile or inflammatory markers from baseline. The orange squares and lines (luseogliflozin group) or black circles and lines (voglibose group) indicate the mean changes and 95% confidence intervals of each marker, relative to the baseline. Each value is represented in Table 2. *MDA-LDL* malondialdehyde low-density lipoprotein, *CRP* C-reactive protein.

Atherogenic lipoproteins decreased after 12 weeks from baseline in both the luseogliflozin and voglibose groups (Fig. 2A,B, Table 2). However, no significant differences were observed in the ratios of the MDA-LDL and small-dense LDL cholesterol concentrations after 12 weeks (relative to baseline levels) between the two groups (the ratios of the mean MDA-LDL and small-dense LDL cholesterol values at week 12 to the baseline values were 1.00 and 0.98 in the luseogliflozin group and 0.99 and 0.91 in the voglibose group [percent change, 0.2% vs. − 0.6% and − 1.7% vs. − 8.6%; p = 0.93 and p = 0.21]).

**Table 2:** Change ratio in biomarkers between the luseogliflozin and voglibose groups.

Variables	Ratio of change from the baseline		p value
	Luseogliflozin group (n = 79)	Voglibose group (n = 78)	
**Atherogenic lipid**			
MDA-LDL, %	0.2 (− 5.6 to 6.3)	− 0.6 (− 7.8 to 7.1)	0.93
Small-dense LDL cholesterol, %	− 1.7 (− 7.4 to 4.3)	− 8.6 (− 15.5 to − 1.1)	0.21
**Inflammatory marker**			
Adiponectin, %	− 1.6 (− 5.4 to 2.4)	− 4.0 (− 9.0 to 1.3)	0.52
High-sensitivity CRP, %	22.5 (− 1.6 to 52.5)	10.0 (− 18.1 to 47.7)	0.55
**Other lipid profile**			
Total cholesterol, %			
Week 4	1.3 (− 1.5 to 4.1)	− 4.4 (− 7.1 to − 1.8)	0.009
Week 12	1.6 (− 2.6 to 5.7)	− 5.3 (− 9.3 to − 1.3)	0.02
Week 24	1.1 (− 2.3 to 4.5)	− 2.0 (− 5.9 to 1.9)	0.19
HDL cholesterol, %			
Week 4	0.2 (− 3.4 to 3.8)	− 5.0 (− 7.4 to − 2.6)	0.02
Week 12	2.4 (− 2.6 to 7.4)	− 4.7 (− 9.1 to − 0.3)	0.04
Week 24	3.4 (− 1.0 to 7.7)	1.4 (− 2.4 to 5.2)	0.43
LDL cholesterol, %			
Week 4	31.5 (− 21.8 to 84.8)	− 3.9 (− 9.2 to 1.4)	0.17
Week 12	13.2 (− 5.4 to 31.9)	− 2.6 (− 8.9 to 3.7)	0.14
Week 24	23.2 (23.0 to 69.3)	− 0.4 (− 6.9 to 5.9)	0.25
Triglyceride, %			
Week 4	− 2.6 (− 11.5 to 7.11)	− 3.2 (− 10.4 to 4.5)	0.65
Week 12	− 1.3 (− 11.7 to 10.3)	− 8.9 (− 17.2 to 0.2)	0.28
Week 24	− 0.3 (− 11.9 to 12.7)	− 7.8 (− 15.3 to 1.27)	0.52

Data are presented as the mean (95% confidence interval).

*CRP* C-reactive protein, *HDL* high-density lipoprotein, *MDA-LDL* malondialdehyde low-density lipoprotein.

In terms of inflammatory markers, different trends were observed for both adiponectin and high-sensitivity CRP levels at 12 weeks after baseline; the adiponectin levels decreased in both the luseogliflozin and voglibose groups, whereas the high-sensitivity CRP levels increased (Fig. 2C,D, Table 2). However, no significant differences were observed in the ratios of adiponectin and high-sensitivity CRP concentrations after 12 weeks (compared with baseline) between the two groups (the ratios of the mean adiponectin and high-sensitivity CRP values at week 12 to the baseline values were 0.98 and 1.22 in the luseogliflozin group and 0.96 and 1.10 in the voglibose group [percent change, − 1.6% vs. − 4.0% and 22.5% vs. 10.0%; p = 0.52 and p = 0.55]).

Time-dependent changes in the high-risk lipid profile and inflammatory markers are listed in Table 3. In the voglibose group, the levels of small dense LDL cholesterol decreased significantly after treatment (relative to baseline), while those of other biomarkers did not. In the luseogliflozin group, no significant change after treatment was observed except for the increase in high-sensitivity CRP.

**Table 3:** Time-dependent changes in biomarker levels.

Variables	Luseogliflozin group (n = 79)					Voglibose group (n = 78)				
	Visit				p value	Visit				p value
	Baseline	Week 4	Week 12	Week 24		Baseline	Week 4	Week 12	Week 24	
Log MDA-LDL	4.50 (0.34)	NA	4.50 (0.33)	NA	0.96	4.50 (0.38)	NA	4.50 (0.35)	NA	0.86
Log small-dense LDL cholesterol	3.33 (0.44)	NA	3.37 (0.42)	NA	0.55	3.37 (0.47)	NA	3.28 (0.47)	NA	0.02
Log adiponectin	2.22 (0.47)	NA	2.20 (0.50)	NA	0.42	2.42 (0.59)	NA	2.38 (0.58)	NA	0.13
Log high-sensitivity CRP	6.65 (1.02)	NA	6.86 (1.13)	NA	0.06	6.65 (1.49)	NA	6.74 (1.50)	NA	0.51
Total cholesterol, mg/dl	175.6 (32.5)	176.4 (32.7)	177.3 (34.9)	175.4 (32.3)	0.93	183.4 (39.1)	174.5 (37.0)	173.4 (34.5)	178.2 (39.7)	0.049
HDL cholesterol, mg/dl	54.8 (15.7)	55.2 (17.7)	54.9 (16.5)	55.7 (16.8)	0.37	56.3 (17.3)	52.3 (15.2)	53.5 (16.4)	56.4 (17.6)	0.90
LDL cholesterol, mg/dl	86.4 (28.4)	89.6 (27.6)	88.1 (28.6)	84.2 (27.5)	0.62	97.5 (32.4)	93.8 (34.5)	93.6 (29.3)	94.8 (32.7)	0.27
Log triglyceride	5.00 (0.52)	4.95 (0.50)	5.00 (0.54)	5.02 (0.56)	0.80	4.85 (0.54)	4.83 (0.53)	4.75 (0.47)	4.79 (0.49)	0.03

Data are presented as the mean (standard deviation). Log-transformed values of MDA-LDL, small-dense LDL cholesterol, adiponectin, high-sensitivity CRP, and triglyceride concentrations are shown.

*CRP* C-reactive protein, *HDL* high-density lipoprotein, *MDA-LDL* malondialdehyde low-density lipoprotein.

### Secondary outcomes

**Figure 3:**
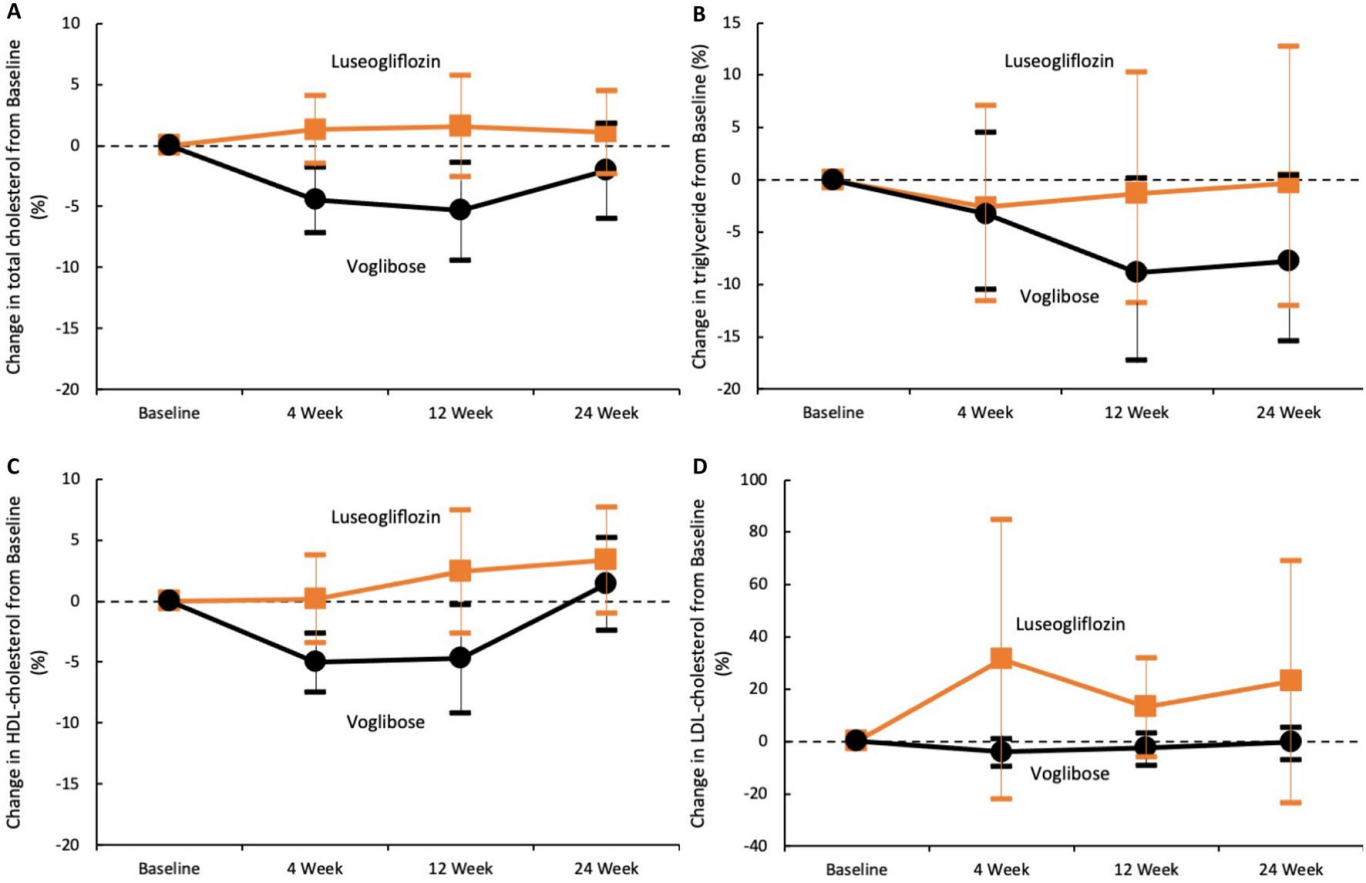
Change in the lipid profile from baseline. The orange squares and lines (luseogliflozin group) or black circles and lines (voglibose group) indicate the mean changes and 95% confidence intervals of each marker, relative to the baseline. Each value is represented in Table 2. *LDL* low-density lipoprotein, *HDL* high-density lipoprotein.

Changes in the lipid profile, including total cholesterol, triglyceride, HDL cholesterol, and LDL cholesterol levels after treatment are shown in Fig. 3 and Table 2. Compared to the baseline levels, the total cholesterol levels after 4 and 12 weeks, and the HDL cholesterol levels after 4 weeks in the voglibose group were significantly lower than those in the luseogliflozin group (percent change, − 4.4% vs. 1.3%, − 5.3% vs. 1.6%, and − 5.0% vs. 0.2%; p = 0.009, p = 0.043, and p = 0.023, respectively). In the luseogliflozin group, LDL cholesterol levels after treatment were greater relative to the baseline but the increase was not statistically significant. Time-dependent changes in the lipid profiles are shown in Table 3. In the voglibose group, the total cholesterol and triglyceride levels decreased significantly after treatment (relative to baseline), while HDL cholesterol levels remained unchanged. In the luseogliflozin group, no significant changes were observed in lipid profile after treatment. No specific adverse events related to the drugs were recorded during the study period.

### Subgroup analyses

There was no significant difference in the atherogenic lipid profiles and inflammatory marker levels after treatment between the luseogliflozin and voglibose groups regardless of the subgroups with prior ASCVD (n = 44 and 47), dyslipidaemia (n = 61 and 59), or statin therapy at baseline (n = 51 and 53; Fig. 1) (Table 4). The time-dependent change (from baseline values) in each parameter after treatment in each patient subgroup was almost similar to that in the overall patient cohort (Supplementary Tables S1–3).

**Table 4:** Change ratio in biomarkers in patient subgroups with prior ASCVD, dyslipidaemia, and statin treatment at baseline.

Variables	Prior ASCVD			Dyslipidaemia			Statin at baseline		
	Change ratio from baseline		p value	Change ratio from baseline		p value	Change ratio from baseline		p value
	Luseogliflozin group (n = 44)	Voglibose group (n = 47)		Luseogliflozin group (n = 61)	Voglibose group (n = 59)		Luseogliflozin group (n = 51)	Voglibose group (n = 53)	
MDA-LDL, %	5.0 (− 3.1 to 13.8)	− 1.0 (− 9.8 to 8.5)	0.45	0.16 (− 5.6 to 6.3)	− 0.6 (− 7.8 to 7.1)	0.64	1.3 (− 5.4 to 8.5)	0.1 (− 8.3 to 9.3)	0.81
Small-dense LDL cholesterol, %	− 0.3 (− 7.8 to 7.8)	− 5.3 (− 15 to 5.5)	0.43	− 1.7 (− 7.4 to 4.3)	− 8.6 (− 15.5 to − 1.1)	0.47	− 5.9 (− 12.4 to 1.1)	− 7.5 (− 16.6 to 2.6)	0.85
Adiponectin, %	− 1.1 (− 6.7 to 4.7)	− 6.7 (− 13.6 to 0.6)	0.43	− 1.6 (− 5.4 to 2.4)	− 4.0 (− 9.0 to 1.3)	0.29	− 0.9 (− 5.6 to 4.2)	− 6.3 (− 12.6 to 0.4)	0.19
High-sensitivity CRP, %	22.0 (− 10.8 to 66.8)	15.2 (− 24.5 to 75.9)	0.83	33.3 (3.0 to 72.4)	21.5 (− 14.3 to 72.3)	0.68	25.4 (− 5.0 to 65.6)	21.4 (− 16.5 to 76.5)	0.93
Total cholesterol, %	2.7 (− 1.7 to 7.1)	− 2.3 (− 5.8 to − 1.2)	0.22	1.6 (− 2.6 to 5.7)	− 5.3 (− 9.3 to − 1.3)	0.09	0.2 (− 3.7 to 4.1)	− 4.7 (− 10.0 to 0.6)	0.24
HDL cholesterol, %	3.5 (− 2.5 to 9.5)	− 2.1 (− 6.5 to 2.3)	0.36	2.4 (− 2.6 to 7.4)	− 4.7 (− 9.1 to − 0.3)	0.09	3.6 (− 1.2 to 8.3)	− 5.0 (− 11.3 to 1.2)	0.053
LDL cholesterol, %	5.9 (− 1.4 to 13.2)	3.5 (− 3.9 to 10.8)	0.94	16.5 (− 6.7 to 39.6)	− 1.9 (− 9.7 to 5.9)	0.14	17.2 (− 10.8 to 45.3)	− 1.4 (− 10.0 to 7.2)	0.29
Triglycerides, %	− 2.2 (− 14.1 to 11.3)	− 11.8 (− 22.1 to − 0.2)	0.25	− 1.3 (− 11.7 to 10.3)	− 8.9 (− 17.2 to 0.2)	0.44	− 8.3 (− 20.5 to 5.8)	− 9.8 (− 20.1 to 1.9)	0.77

Data are presented as the mean (95% confidence interval).

*ASCVD* atherosclerotic cardiovascular disease, *CRP* C-reactive protein, *HDL* high-density lipoprotein, *MDA-LDL* malondialdehyde low-density lipoprotein.

## Discussion

The present study presents a prespecified sub-analysis of the MUSCAT-HF study to compare the effects of an SGLT2 inhibitor and an alpha-glucosidase inhibitor on atherogenic lipoproteins (MDA-LDL and small-dense LDL cholesterol) and inflammatory markers (adiponectin and high-sensitivity CRP), as related to the incidence of ASCVD in diabetes patients with heart failure. No significant differences were observed in the levels of the atherogenic lipoproteins after treatment (relative to the baseline) between the two groups, while small-dense LDL cholesterol levels decreased significantly after the initiation of voglibose. Similarly, the levels of inflammatory markers in the luseogliflozin group did not improve significantly after treatment compared to those in the voglibose group. The lipid profile (including total cholesterol, triglyceride, HDL cholesterol, and LDL cholesterol) did not differ significantly between the two groups after treatment, whereas total cholesterol and triglyceride levels decreased significantly after the initiation of voglibose. Among the patient subgroups with prior ASCVD, dyslipidaemia, and statin at baseline, the time-dependent changes in each parameter after treatment were not significantly different between the two groups.

### SGLT2 inhibitors and atherogenic lipid profile

Several reports have shown that SGLT2 inhibitors can reduce the serum levels of total cholesterol and triglyceride^15,25^, although whether SGLT2 inhibitor treatment can reduce the serum levels of HDL cholesterol and LDL cholesterol remains controversial. In an observational study, Cha et al. found that LDL cholesterol levels significantly increased after an SGLT2 inhibitor was used as add-on therapy, when compared with the effects of dipeptidyl-peptidase IV inhibitors in diabetes patients treated with metformin or sulfonylurea^26^. Furthermore, in an RCT conducted on patients with diabetes, Schernthaner et al. found that canagliflozin treatment significantly increased serum LDL cholesterol levels compared with sitagliptin^27^. However, few studies have been conducted to investigate the effect of SGLT2 inhibitors on high-risk lipid profiles, especially oxidative LDL. In a single centre RCT conducted in patients with diabetes, Hayashi et al. reported that dapagliflozin significantly reduced small-dense LDL cholesterol levels after treatment compared with those at baseline^15^. Since both LDL cholesterol and large buoyant LDL levels were significantly higher after treatment than at baseline, the mechanism whereby SGLT2 inhibitors increased LDL levels was suggested to involve suppressed conversion of cholesterol-rich, large buoyant LDL to cholesterol-poor small-dense LDL cholesterol. In this study, the changes in MDA-LDL and small-dense-LDL cholesterol levels after SGLT2 treatment were not significantly different from baseline. The LDL cholesterol levels after SGLT2 treatment increased, but they did not reach statistical significance. Unlike previous studies, this study was based on the original RCT including stable diabetes patients with heart failure, and therefore, the baseline lipid profiles were in the normal range. A lower risk-lipid profile at baseline might reduce the impact of SGLT2 inhibitors on lowering serum lipid levels. We believe that the inconsistent results among different studies investigating the effects of SGLT2 inhibitors on the lipid profile, including those reported herein, might be attributable to study participants than to different drugs used.

In clinical trials, SGLT2 inhibitors significantly suppressed the incidence of cardiovascular death, especially in patients with prior ASCVD, but not in high-risk patients without prior ASCVD^1–3^. Additionally, SGLT2 inhibitors showed no significant impact on individual events, such as myocardial infarction and stroke, except for hospitalisation for heart failure. This result may reflect the marginal effect of SGLT2 inhibitors on dyslipidaemia.

### Voglibose and lipid metabolism

In our study, while no significant differences were observed in the lipid profiles between the luseogliflozin and voglibose groups, small-dense LDL cholesterol, total cholesterol, and triglyceride levels decreased significantly after the initiation of voglibose. Alpha-glucosidase inhibitors, including voglibose, are used to treat diabetes; they lower glucose levels and reversibly inhibit the absorption of complex carbohydrates. Several studies have also reported triglyceride and total cholesterol lowering effects of alpha-glucosidase^28–30^. The findings of our study are consistent with these results. The mechanism of voglibose action underlying the decrease in the levels of small-dense LDL cholesterol remains unclear, although a hypothesis has been proposed. Studies have shown that an increase in small-dense LDL cholesterol levels is associated with metabolic syndrome or insulin resistance, and positively correlates to triglyceride level^31,32^. Therefore, voglibose may indirectly reduce small-dense LDL cholesterol levels by lowering triglyceride levels and thereby improving dyslipidaemia.

### Association of SGLT2 inhibitors with inflammatory markers

Previously, Garvey et al. reported the positive effects of SGLT2 inhibitor on inflammatory markers in patients with type 2 diabetes^33^. They reported that canagliflozin treatment significantly increased the serum levels of adiponectin and tumour necrosis factor ɑ when compared with glimepiride treatment. The serum levels of interleukin-6 and CRP after treatment were also reduced; however, statistical significance was achieved for interleukin-6, but not for CRP. In this study, the changes in serum adiponectin and high-sensitivity CRP levels were not significantly different before and after treatment. We speculate that differences in the study participants between the previous study and the present study may have resulted in different findings. The patients in the previous study had a higher risk for ASCVD, such as obesity (mean body weight: over 90 kg and mean body mass index: over 30), than the patients in this study. In the previous study, for instance, the baseline serum level of adiponectin was markedly lower than that in this study. Furthermore, the serum level of adiponectin before luseogliflozin treatment in this study was higher than that after canagliflozin treatment in the previous study. We think the effect of SGLT2 inhibitors on inflammatory markers might be inadequate in patients with low cardiovascular risk at baseline. However, in this study, the high-sensitivity CRP levels remained higher (relative to baseline) after the initiation of luseogliflozin although the increase was not statistically significant. The underlying mechanism remains unclear, and thus, further investigation of SGLT2 inhibitors and inflammation is warranted.

### Effect of SGLT2 inhibitors on ASCVD

In previous clinical studies^1–6^, SGLT2 inhibitors showed consistent positive effects on renal outcomes and reducing heart failure hospitalisation, regardless of the clinical background of the patients (i.e., with or without diabetes or high ASCVD risk). The effect of SGLT2 inhibitors on cardiovascular death or the incidence of ASCVD (including myocardial infarction and stroke) are controversial. The results of this study suggest that SGLT2 inhibitors might have an inadequate effect on atherogenic lipid profiles or inflammatory markers in patients with a low risk of cardiovascular disease (including well or appropriate control of glucose levels, obesity, and fatty liver). Therefore, a poor positive effect of SGLT2 inhibitors on atherogenic risk factors could explain its inconsistent results for ASCVD. The findings of this study support the fact that to prevent the incidence of myocardial infarction and stroke caused by atherogenic lipids or inflammation, sufficient additional medication such as metformin or a statin (with protective effects) and not treatment with SGLT2 inhibitors alone, is necessary for patients with diabetes.

Apart from the RCT subanalysis, this study has several limitations. First, the study population was comparatively small, and thus, these results should be interpreted with caution as they are exploratory in nature. Second, this study included stable diabetes patients with heart failure irrespective of dyslipidaemia status. As a result, the baseline lipid profile and inflammatory markers were almost within the normal range. This might have led to underestimation of the effects exerted by luseogliflozin on outcome variables. Third, the outcomes assessment was performed only 4-, 12-, and 24 weeks after the initiation of the target drug. This design was acceptable for the main analysis of the RCT, wherein short-term effects of the target drugs were assessed on the change in B-type natriuretic peptide levels; however, the medication period might not be sufficient to assess drug effects on the lipid profile and inflammation, as these parameters change dynamically upon long-term drug use. Fourth, luseogliflozin is only approved in Japan and not in other countries. Furthermore, this study included only Japanese patients. Thus, the generalizability of these results is limited. Finally, the baseline covariates including high-risk lipid profiles and inflammatory markers between luseogliflozin and voglibose groups were well-balanced. However, the randomisation process was optimized for the main analysis of the RCT. Therefore, residual and unmeasured confounding factors between study drugs and the outcomes might have remained even after adjusting for stratified factors of randomisation. We hope to clarify whether SGLT2 inhibitors reduce high-risk lipid levels and inflammation through a larger-scale and well-designed prospective study (multicentred, placebo-controlled, and randomised trial) in the future.

## Conclusions

In the subanalysis using the RCT data of patients with type 2 diabetes and heart failure, the SGLT2 inhibitor (luseogliflozin) did not significantly reduce the serum levels of MDA-LDL or small-dense LDL cholesterol after treatment when compared with the ɑ-glucosidase inhibitor, voglibose. Similarly, no significant improvement was observed in inflammatory markers (including adiponectin and high-sensitivity CRP) after luseogliflozin treatment. Our findings may bear implications on the effects of SGLT2 inhibitors on risk factors beyond their known impact and suggest the necessity of future studies for complete elucidation of the mechanism of drug action.

## Supplementary Information

Supplementary Information 1.

Supplementary Information 2.

## Data Availability

The datasets generated and/or analysed during the current study are not publicly available due to prohibition by the Ethics Committee, but are available from the corresponding author on reasonable request.

## Abbreviations

ASCVD: Atherosclerotic cardiovascular disease
BNP: B-type natriuretic peptide
CRP: C-reactive protein
eGFR: Estimated glomerular filtration rate
HbA1C: Haemoglobin A1c
HDL: High-density lipoprotein
LDL: Low-density lipoprotein
MDA-LDL: Malondialdehyde low-density lipoprotein
MUSCAT-HF: Management of diabetic patients with chronic heart failure and preserved left
SGLT2: Sodium-glucose cotransporter 2
UMIN-CTR: University Hospital Medical Information Network Clinical Trial Registry
