# Supplementary material for: Effects of luseogliflozin and voglibose on high-risk lipid profiles and inflammatory markers in diabetes patients with heart failure
Source: Sci Rep. 2022 Sep 14;12:15449. doi: 10.1038/s41598-022-19371-6 (PMC9474821; doi:10.1038/s41598-022-19371-6)
Supplement: Supplementary file 1 — Supplementary Information 1. [file 41598_2022_19371_MOESM1_ESM.docx]

**Study protocol**

- Study organization
- Study protocol
- Data A1. Study protocol
- Data A2. Laboratory testing
- Data A3. Outcome definitions for adverse events
- **Study organization**

**The investigators, clinical coordinators, and institutions involved in the MUSCAT-HF study:**

Kentaro Ejiri (Tamano City Hospital and Okayama University Graduate School of Medicine, Density and Pharmaceutical Sciences), Toru Miyoshi, Kazufumi Nakamura and Hiroshi Ito (Okayama University Graduate School of Medicine, Density and Pharmaceutical Sciences); Hajime Kihara (Kihara Cardiovascular Clinic); Yoshiki Hata (Minamino Cardiovascular Hospital); Toshihiko Nagano (Iwasa Hospital); Atsushi Takaishi (Mitoyo General Hospital); Hironobu Toda (Okayama East Neurosurgery Hospital and Okayama University Graduate School of Medicine, Density and Pharmaceutical Sciences); Seiji Namba (Okayama Rosai Hospital); Yoichi Nakamura (Specified Clinic of Soyokaze Cardiovascular Medicine and Diabetes Care); Satoshi Akagi (Akaiwa Medical Association Hospital and Okayama University Graduate School of Medicine, Density and Pharmaceutical Sciences); Satoru Sakuragi (Iwakuni Clinical Center); Taro Minagawa (Minagawa Cardiovascular Clinic); Yusuke Kawai (Okayama City Hospital); Nobuhiro Nishii (Yoshinaga Hospital and Okayama University Graduate School of Medicine, Density and Pharmaceutical Sciences); Tetsuya Sato and Soichiro Fuke (Japanese Red Cross Okayama Hospital); Masaki Yoshikawa and Hiroyasu Sugiyama (Fukuyama City Hospital); Michio Imai (Imai Heart Clinic); Naoki Gotoh (Gotoh Clinic); Tomonori Segawa (Asahi University Hospital); Toshiyuki Noda (Gifu Prefectural General Medical Center); and Masatoshi Koshiji (Gifu Seiryu Hospital)

**Steering Committee:**

Hiroshi Ito (Chair), Kazufumi Nakamura, Toru Miyoshi, Kentaro Ejiri (Okayama University Graduate School of Medicine, Density and Pharmaceutical Sciences, Okayama, Japan); Satoru Sakuragi (Iwakuni Clinical Center, Yamaguchi, Japan); Mitsuru Munemasa (Okayama Medical Center, Okayama, Japan); Seiji Nanba (Okayama Rosai Hospital, Okayama, Japan); Tomosato Suezawa (Takamatsu Red Cross Hospital, Kagawa, Japan); and Atsushi Takaishi (Mitoyo General Hospital, Kagawa, Japan)

**Statistical consulting:**

Tetsutaro Hamano (P4 Statistics Co. Ltd., Tokyo, Japan)

**Ethics Committee:**

The Okayama University Hospital Ethics Committee (representative); The Tamano City Hospital Ethics Committee; The Okayama University Hospital Ethics Committee; The Mitoyo General Hospital Ethics Committee; The Okayama Rosai Hospital Ethics Committee; The Iwakuni Clinical Center Ethics Committee; The Okayama City Hospital Ethics Committee; The Japanese Red Cross Okayama Hospital Ethics Committee; and The Fukuyama City Hospital Ethics Committee. The study was also approved by The Okayama University Hospital Ethics Committee in other participating centres which did not organize own ethics committee.

**Clinical Event Committee:**

Masayuki Doi (Kagawa Prefectural Central Hospital, Kagawa, Japan); Takefumi Oka (Tsuyama Central Hospital, Okayama, Japan)

**Data Monitoring**

Given that invasive intervention will not be performed in this study, data monitoring is not planned.

- **Study protocol**

**Data A1. Study protocol**

**Prospective Comparison of Luseogliflozin and Alpha- glucosidase on the Management of Diabetic Patients with Chronic Heart Failure and Preserved Ejection Fraction**

Brier title: MUSCAT-HF study

First date of disclosure of the study information: July 14, 2015

Final date of disclosure of the study information: April 27, 2018

Funding source: This study was funded by Novartis Pharma K.K. It was not involved in the design of the protocol, the conduct of the study, or the analyses or reporting of the data.

A person in charge:

Hiroshi Ito

Department of Cardiovascular Medicine

Okayama University Graduate School of Medicine, Dentistry and Pharmaceutical Sciences

Address: 2-5-1 Shikata-cho, Kita-ku, Okayama, Japan

TEL: (+81) 86-235-7349

E-MAIL: [itomd@md.okayama-u.ac.jp](mailto:itomd@md.okayama-u.ac.jp)

Contents

I. Summary of study plan

II. Background of study plan

III. Study plan

1. Purpose

2. Study population

3. Consent

4. Interventions

5. Methods

6. Endpoints

7. Cancellation and withdrawal

8. Study period

9. Statistics

10. Data management and analysis

11. Ethical consideration

12. Methods of plan change

13. A person in charge and office

14. Funding sauce

15. Collaboration with other scientists or research institutions

16. References

**I. Summary of study plan**

The MUSCAT-HF trial was a multi-centre, prospective, open-label, randomized controlled trial to assess the effect of luseogliflozin compared with voglibose on left ventricular load in patients with type 2 diabetes mellitus (T2DM) and heart failure with preserved ejection fraction (HFpEF).

**II. Background of study plan**

Recent randomized controlled trials showed that sodium glucose cotransporter 2 (SGLT2) inhibitors reduced all-cause mortality, cardiovascular mortality, and hospitalization of heart failure in type 2 diabetes compared with placebo. These results indicated that SGLT2 inhibitors may be effective in lowering glucose levels and reducing cardiovascular events, particularly in patients with heart failure. Given that these trials were not specifically designed to investigate the effect of SGLT2 inhibitors in heart failure patients, no detailed data on their effects in heart failure were obtained.

**III. Study plan**

1. Purpose

The aim of this study was to evaluate the efficacy of luseogliflozin, an SGLT2 inhibitor, compared with voglibose, an alpha-glucosidase inhibitor, using brain natriuretic peptide (BNP) as the index of therapeutic effect in patients with T2DM and HFpEF. The results of this study will support a novel strategy for the treatment of heart failure using an SGLT2 inhibitor, independent of its glucose-lowering effects.

2. Study population

The planned sample size of this study was 95 patients per group (190 patients in total). The recruitment of study patients was planned to take place from September 2015 to September 2018. Patients aged ≥20 years with T2DM (haemoglobin A1c [HbA1C] ≤9.0%) and HFpEF (left ventricular ejection fraction ≥45%) needing additional treatment for T2DM despite the ongoing treatment are eligible for participation. The key inclusion and exclusion criteria are detailed in Table 1. Given that the definition of chronic heart failure according to European Society of Cardiology guidelines includes BNP ≥35 pg/ml, patients with BNP <35 pg/ml was excluded from this study. Study candidates were assessed for eligibility within 4 weeks prior to enrolment.

Inclusion and exclusion criteria

| Inclusion criteria |
| --- |
| 1) Diagnosis of T2DM and left ventricular ejection fraction >45% with current or previous symptoms of heart failure (dyspnoea on effort, orthopnoea, or leg oedema) |
| 2) Inadequately controlled T2DM in patients who have received diet and exercise therapy, a lifestyle modification program, and hypoglycaemic medications based on standard guidelines of the Japan Diabetes Society |
| 3) Age >20 years |
| 4) Provision of written informed consent prior to participation |
| Exclusion criteria |
| 1) BNP <35 pg/ml |
| 2) Use of alpha-glucosidase inhibitors, SGLT2 inhibitors, glinides, or high-dose sulfonylurea |
| 3) Renal insufficiency (eGFR <30 ml/min/1.73m^2^) |
| 4) Left ventricular ejection fraction <45% |
| 5) History of severe ketoacidosis or diabetic coma within 6 months prior to participation |
| 6) Serious infection or severe trauma, or perioperative patients |
| 7) Type 1 diabetes mellitus |
| 8) Poorly controlled T2DM (HbA1c >9.0%) |
| 9) Uncontrolled hypertension (systolic blood pressure >160 mmHg) |
| 10) History of stroke, myocardial infarction, or severe cardiovascular disease with hospitalization within 6 months prior to participation |
| 11) Women who are pregnant or breastfeeding |
| 12) Allergy to either investigation product |
| 13) Other medical reason at the investigator’s discretion |

T2DM, type 2 diabetes mellitus; BNP, brain natriuretic peptide; SGLT2, sodium/glucose cotransporter 2; eGFR, estimated glomerular filtration rate; HbA1C, haemoglobin A1C

3. Consent

1) Written informed consent is needed to be received.

2) If patients do not have ability of judgment, informed consent cannot be received.

Informed Consent Form

1 Purpose

2 Efficacy and side effect

3 Alternative treatment options

4 NO disadvantage by rejection

5 Withdrawal rights

6 Ethics

4. Interventions

1) Voglibose: subjects who receive voglibose (2.5 mg once daily)

2) Luseogliflozin: subjects who receive luseogliflozin (2.5 mg once daily)

5. Methods

Patients fulfilling all criteria who provide written informed consent to participate in this study were enrolled and subsequently randomized (1:1) to receive luseogliflozin (2.5 mg once daily) or voglibose (0.2 mg three times daily) in addition to their background medication. Randomization was performed using a computer-generated random sequence web response system. Patients were stratified by age (<65 years, ≥65 years), baseline HbA1c (<8.0%, ≥8.0%), baseline BNP (<100 pg/ml, ≥100 pg/ml), baseline renal function (eGFR ≥60 ml/min/1.73 m2, <60 ml/min/1.73 m2), use of thiazolidine or not, and presence or absence of atrial fibrillation (AF) and flutter (AFL) at screening.

Assessments during the study period are listed in Figure 1. Laboratory data, electrocardiogram, echocardiography and patients’ vital signs, body weight, and waist circumference, were evaluated at 4 ± 2 weeks (visit 29 ± 14 days) and 12 weeks (visit 85 ± 28 days) after initiation of study treatment. Safety and tolerability were assessed during the treatment period. The primary outcome of change in BNP compared with baseline was evaluated at 12 weeks (visit 85 ± 28 days) and patients were followed up for an additional 12 weeks (visit 169 ± 28 days) after the end of treatment. If a patient’s glycaemic control worsens after 4 ± 2 weeks, the investigator could increase the dose of allocated treatment (to luseogliflozin 5 mg once daily or voglibose 0.3 mg three times daily) and other specific T2DM drugs, except for sulfonylureas. Investigators were also encouraged to treat all other cardiovascular risk factors according to local standard of care. Under the following circumstances, the investigator must evaluate the data and patient’s vital sign: 1) discontinuation of study treatment; 2) dose increase of specific treatment for heart failure; 3) initiation of new treatment for heart failure; 4) withdrawal from the study. The permitted medications for the treatment of heart failure included angiotensin-converting enzyme inhibitors, angiotensin receptor blockers, beta-blockers, diuretics, and mineralocorticoid/aldosterone receptor antagonists.

6. End points

Assessments during the study period are listed in Figure 2.

Primary outcome

The primary outcome of this study was the difference in BNP after 12 weeks (visit 85±28 days) of treatment between the luseogliflozin and the voglibose groups, defined as the difference in logarithmic BNP change calculated as follows:

(*) BNP proportional change = BNP (at follow-up)/ BNP (at baseline),

(†) Logarithmic BNP change = logarithmic BNP (at follow-up) – logarithmic BNP (at baseline),

In other words, (*) = exponential (†).

Furthermore, we calculated the ratio of BNP change rate

(‡) The ratio of BNP proportional change (the luseogliflozin group to the voglibose group) = (*) (in the luseogliflozin group)/ (*) (in the voglibose group),

(§) The difference of logarithmic BNP change = (†) (in the luseogliflozin group) – (†) (in the voglibose group),

In other words, (‡) = exponential (§)

Secondary outcomes

The key secondary outcomes of this study were the differences in the following parameters between the luseogliflozin and the voglibose groups:

1) Ratio of early mitral inflow velocity to mitral annular early diastolic velocity (E/e')

2) Left ventricular ejection fraction

3) Body weight

4) HbA1c

The difference in E/e' and HbA1c between the groups was defined as the difference in logarithmic E/e' and HbA1c using the same calculation as for BNP. The difference in body weight and left ventricular ejection fraction was defined as the difference between those parameters at follow-up and at baseline. Further exploratory analysis is listed in exploratory analysis section.

Safety outcomes: including, but not limited to:

• clinical laboratory tests, vital signs, 12-lead electrocardiogram (ECG), physical examination, and the use of rescue medication

• Adverse events including major adverse cardiovascular events (MACE), hypoglycaemic adverse events (requiring any intervention), and urinary tract infection.

Safety was assessed based on adverse events reported throughout the study, clinical laboratory tests, vital signs, 12-lead electrocardiogram, physical examination, and the use of rescue medication. Prespecified adverse events included MACE, hypoglycaemic adverse events (requiring any intervention), and urinary tract infection (details listed in Outcome definitions for adverse events section)

7. Cancellation and withdrawal

Discontinuance criteria

| Withdrawal criteria | 1) Inadequate glycaemic control after administration of the study drug |
| --- | --- |
|  | 2) Suspect of adverse side effects of the study drug |
|  | 3) Frequent hypoglycaemia |
|  | 4) Onset of adverse cardiovascular event† |
|  | 5) Declaration of withdrawal from the study by the participant |
|  | 6) Turning out of misunderstanding of all criteria for eligibility after enrolment |
|  | 7) Pregnancy after enrolment |
|  | 8) Lower adherence for administration of the study drug (< 70%) |
|  | 9) Assessment of inadequate for the study by the attending doctor |
|  |  |
| †Cardiovascular event | 1) Addition of heart failure treatment drugs as follows; |
|  | angiotensin-converting enzyme (ACE) inhibitors, angiotensin receptor blockers (ARB), beta-blockers, diuretics, and aldosterone antagonists |
|  | 2) Hospitalization of heart failure |

8. Study period

Between December 1, 2015, and March 31, 2019

9. Statistics

Sample size and power calculation

The primary hypothesis of this study was that the SGLT2 inhibitor luseogliflozin could reduce cardiac load in patients with T2DM and HFpEF. Therefore, the primary outcome was the difference in change in BNP from baseline to 12 weeks between patients receiving luseogliflozin or voglibose. As of the start of recruitment in September 2015, no interventional study of the effect of SGLT2 inhibitors on heart failure in patients with T2DM had been reported. Therefore, we had estimated that BNP change rate in the luseogliflozin group would be 30% lower as compared with that in the globose group according to previous studies of the effect of renin-angiotensin-aldosterone system inhibitors on heart failure ^15-17^. The standard deviation of the natural logarithmic transformation of BNP was estimated at 0.83, in reference to the PARAMOUNT study ^17^. A minimum of 172 patients (86 patients per group) is required to provide 80% power with a two-sided ɑ level of 0.05 by Student’s t-test on the ratio of BNP change rate between the luseogliflozin and voglibose groups. With 10% of patients estimated to withdraw from participation during the study period, the final enrolment target was set at 190 patients (95 patients per group).

Analysis plan

In the efficacy analysis, the primary population comprised the Full Analysis Set (FAS), defined as all randomized patients who received one dose of study drug and were followed up at least once. Patients with no BNP data and patients who withdrew or discontinue treatment was excluded from the FAS. Missing values at 4, 12, and 24 weeks were replaced by the last observed value for that variable (last observation carried forward). In the primary outcome analysis, baseline observation carried forward analysis was also performed. Efficacy analysis was performed according to the treatment to which patients are randomly assigned, based on the intention-to-treat analysis. The primary outcome analysis was based on an analysis of covariance (ANCOVA) (α = 0.05, level of significance) for the ratio of BNP change rate in the FAS. Adjusted covariates included the assigned treatment (luseogliflozin, voglibose), baseline age (<65 or ≥65 years), baseline HbA1c (<8.0 or ≥8.0%), baseline BNP (<100 or ≥100 pg/ml), baseline renal function (eGFR ≥60 or <60 ml/min/1.73 m2), use of thiazolidine or not at baseline, and presence or absence of AF and AFL at baseline as stratified factors of randomization. Furthermore, BNP change rate, ratio of BNP change rate, and 95% confidence intervals were calculated. The same ANCOVA analysis as for the primary outcome was performed for the ratio of BNP change rate at 4 weeks and 24 weeks between the two groups.

Prespecified subgroup analyses were performed on the primary outcome using ANCOVA (covariates: assigned treatment and BNP at screening) in the following subgroups: baseline age (<65 or ≥65 years), baseline HbA1c (<8.0 or ≥8.0%), baseline BNP (<100 or ≥100 pg/ml), baseline renal function (eGFR ≥60 or <60 ml/min/1.73 m2), use of thiazolidine or not at baseline, baseline body weight (<60 kg, ≥60 kg), and presence or absence of AF and AFL at baseline. Furthermore, exploratory analysis on the primary outcome was performed in subgroups based on blood pressure, heart rate, waist circumference, cardiovascular risk factors (hypertension, T2DM, hyperuricemia, family history, and smoking), alcohol consumption, regular medication, and serum lipid levels (details listed in exploratory analysis section).

The key secondary outcomes, difference in E/e', left ventricular ejection fraction, body weight, and HbA1C at 12 weeks between the luseogliflozin and voglibose groups, were analysed using the same ANCOVA as for the primary outcome. Subgroup analysis for the key secondary outcomes was performed in the same subgroups as for the primary outcome analysis. The following secondary outcomes was also analysed using the same analysis plan: E/e', left ventricular ejection fraction, body weight, and HbA1C at 4 and 24 weeks; and exploratory parameters at 4, 12, and 24 weeks.

For the safety analysis, the primary population was the Safety Analysis Set (SAFETY), defined as all patients who receive at least one dose of study drug. Although patients who withdrew without receiving study drug will be excluded from SAFETY, other patients who withdrew for any other reason was included. The safety analysis was performed according to the treatment administered to patients in practice, based on the as-treated analysis. Analysis of SAEs (MACE, hypoglycaemia, and urinary tract infection) was performed using the Cochran–Mantel–Haenszel test with stratification factors of age (<65 or ≥65 years), baseline HbA1c (<8.0 or ≥8.0%), baseline BNP (<100 or ≥100 pg/ml), baseline renal function (eGFR ≥60 or <60 ml/min/1.73 m2), use of thiazolidine or not, and presence or absence of AF and AFL at screening.

All comparisons were planned, and the analyses was two sided With P values <0.05 considered statistically significant. All statistical analyses were performed using IBM SPSS Statistics 24 (IBM, Armonk, NY) and Stata/SE 15.1 for Mac (StataCorp, College Station, TX). The statistical analysis plan was developed by the principal investigator and a biostatistician prior to the completion of patient recruitment and database lock.

Exploratory analysis

Further exploratory analysis in this study was planned for such parameters.

1. Blood glucose
2. Lipid metabolism [total cholesterol, high density lipoprotein, triglyceride, small dense low-density lipoprotein and Malondialdehyde-modified low density lipoprotein]
3. Blood pressure
4. High-sensitive CRP
5. Adiponectin, microalbuminuria
6. Urinary 8-hydroxy-2' –deoxyguanosine
7. Estimated GFR

10. Data management and analysis

Members of the Steering Committee also designed the study and were responsible for its conduction (details listed in Study organization section). Significant adverse events (SAEs) occurring within 30 days after final administration of the study drug or after 30 days with a suspicion of association with the study drug, as well as all pregnancies, was immediately reported to the Steering Committee and the sponsor by the investigator, in accordance with GCP.

11. Ethical consideration

All participants provided written informed consent before enrolling.

This study was conducted according to the principles expressed in the Declaration of Helsinki.

This study was approved by the Okayama University Graduate School of Medicine, Density and Pharmaceutical Sciences and the Okayama University Hospital Ethics Committee, as well as the ethics committee of each participating centre. Trial registration: UMIN Clinical Trials Registry (UMIN-CTR), UMIN000018395, https://upload.umin.ac.jp/cgi-open-bin/ctr_e/ctr_view.cgi?recptno=R000021301

12. Methods of plan change

When needed, investigators made a discussion and decision of plan change.

13. A person in charge and office

Hiroshi Ito

Department of Cardiovascular Medicine

Okayama University Graduate School of Medicine, Dentistry and Pharmaceutical Sciences

Address: 2-5-1, Shikata-cho, Kita-ku, Okayama

TEL: (+81) 86-235-7349

[itomd@md.okayama-u.ac.jp](mailto:itomd@md.okayama-u.ac.jp)

14. Funding sauce

This study was funded by Novartis Pharma K.K. It was not involved in the design of the protocol, the conduct of the study, or the analyses or reporting of the data.

15. Collaboration with other scientists or research institutions

1) Measurement institutions:

SRL Hachioji laboratory

Hachioji Komiya 51, Tokyo 192-8535

2) Statistical consulting:

Tetsutaro Hamano (P4 Statistics Co. Ltd., Tokyo, Japan)

16. References

15. Rousseau MF, Gurne O, Duprez D, Van Mieghem W, Robert A, Ahn S, Galanti L, Ketelslegers JM. Beneficial neurohormonal profile of spironolactone in severe congestive heart failure: Results from the rales neurohormonal substudy. *J Am Coll Cardiol*. 2002;40:1596-1601

16. Tsutamoto T, Wada A, Maeda K, Mabuchi N, Hayashi M, Tsutsui T, Ohnishi M, Sawaki M, Fujii M, Matsumoto T, et al. Effect of spironolactone on plasma brain natriuretic peptide and left ventricular remodeling in patients with congestive heart failure. *J Am Coll Cardiol*. 2001;37:1228-1233

17. Solomon SD, Zile M, Pieske B, Voors A, Shah A, Kraigher-Krainer E, Shi V, Bransford T, Takeuchi M, Gong J, et al. The angiotensin receptor neprilysin inhibitor lcz696 in heart failure with preserved ejection fraction: A phase 2 double-blind randomised controlled trial. *Lancet*. 2012;380:1387-1395

**Data A2. Laboratory testing**

Brain natriuretic peptide, N-terminal brain natriuretic peptide, adiponectin, small dense low-density lipoprotein, malondialdehyde-modified low density lipoprotein, high-sensitive C-reactive protein, microalbuminuria, urinary 8-hydroxy-2' –deoxyguanosine

These parameters were measured in a central laboratory (SRL, Inc. Hachioji, Tokyo, Japan).

White blood cell, red blood cell, platelet, haemoglobin, haematocrit, aspartate aminotransferase, alanine aminotransferase, lactate dehydrogenase, blood urea nitrogen, serum creatinine, uric acid, serum sodium, serum potassium, serum chloride, total cholesterol, high density lipoprotein, triglyceride, total protein, albumin, blood sugar, glycohemoglobin

These parameters were measured in each institution.

**Data A3. Outcome definition of adverse events**

Major adverse cardiovascular events (MACE) include cardiovascular death, acute coronary syndrome, hospitalization of heart failure, and stroke.

- Cardiovascular death

The cause of death will be determined by the principal condition that caused the death, not the immediate mode of death. Clinical Events Committee (CEC) members will review all available information and use their clinical expertise to adjudicate the cause of death. All deaths not attributed to the categories of cardiovascular (CV) death and not attributed to a non-CV cause are presumed CV deaths and are part of the CV mortality outcome. Death certificates or summaries, if possible, including the date of death and other relevant details, will be provided for all patients who have died. However, if a death certificate is the only information available for review in addition to the patient data in the clinical trial database, the CEC may decide not to use this information as cause of death if another aetiology appears more plausible. The following definitions will be used for the adjudication of fatal cases:

Sudden cardiac death. Death that occurs unexpectedly in a previously stable patient and includes the following:

• Witnessed and instantaneous death without new or worsening symptoms

• Witnessed death within 60 minutes of the onset of new or worsening cardiac symptoms

• Witnessed death attributed to an identified arrhythmia (e.g., captured by electrocardiogram or witnessed on a monitor by either a medic or paramedic)

• Subject unsuccessfully resuscitated from cardiac arrest or successfully resuscitated from cardiac arrest that dies within 24 hours without identification of a non-cardiac aetiology

• Un-witnessed death with no conclusive evidence of another, non-CV, cause of death (i.e., presumed CV death).

Sudden death attributable to acute myocardial infarction (MI) (MI type 3). Sudden death occurring up to 14 days after a documented acute MI (verified either by the diagnostic criteria outlined for acute MI or by autopsy findings showing recent MI or recent coronary thrombus) where there is no conclusive evidence of another cause of death. If death occurs before the biochemical confirmation of myocardial necrosis can be obtained, adjudication should be based on clinical presentation and ECG evidence.

Death attributable to heart failure or cardiogenic shock. Death occurring in the context of clinically worsening symptoms and/or signs of congestive heart failure (CHF) without evidence of another cause of death.

New or worsening signs and/or symptoms of CHF include any of the following:

• New or increasing symptoms and/or signs of heart failure requiring the initiation of, or an increase in, treatment directed at heart failure or occurring in a patient already receiving maximal therapy for heart failure

• Heart failure symptoms or signs requiring continuous intravenous therapy or oxygen administration

• Confinement to bed predominantly because of heart failure symptoms

• Pulmonary oedema sufficient to cause tachypnoea and distress not occurring in the context of an acute MI or as the consequence of an arrhythmia occurring in the absence of worsening heart failure

• Cardiogenic shock not occurring in the context of an acute MI or as the consequence of an arrhythmia occurring in the absence of worsening heart failure

– Cardiogenic shock is defined as systolic blood pressure (SBP) <90 mmHg for more than 1 hour, ack of response to fluid resuscitation and/or heart rate correction, and judged to be secondary to cardiac dysfunction and associated with at least one of the following signs of hypoperfusion:

1. Cool, clammy skin
2. Oliguria (urine output <30 mL/hour)
3. Altered sensorium
4. Cardiac index <2.2 L/min/m^2^

Cardiogenic shock can also be defined in the presence of SBP ≥90 mmHg or for a time period <1 hour if the blood pressure measurement or time period is influenced by the presence of positive inotropic or vasopressor agents alone and/or with mechanical support <1 hour. The outcome of cardiogenic shock will be based on CEC assessment and must occur after randomization. Episodes of cardiogenic shock occurring before and continuing after randomization will not be part of the study outcome. This category will include sudden death occurring during an admission for worsening heart failure

Death attributable to stroke or cerebrovascular event. Death occurring up to 30 days after a stroke that is either attributable to the stroke or caused by a complication of the stroke.

Death attributable to other CV causes. Death must be caused by a fully documented CV event not included in the above categories (e.g., dysrhythmia, pulmonary embolism, or CV intervention). Death attributable to an MI that occurs as a direct consequence of a CV investigation/procedure/operation will be classified as death due to another CV cause.

Non-CV death

Non-CV death is defined as any death not covered by cardiac death or vascular death. The CEC will be asked to determine the most likely cause of non-CV death. Examples of non-CV death are pulmonary causes, renal causes, gastrointestinal causes, infection (including sepsis), non-infectious causes (e.g., systemic inflammatory response syndrome), malignancy (i.e., new malignancy, worsening of prior malignancy), haemorrhage (not intracranial), accidental/trauma, suicide, non-CV organ failure (e.g., hepatic failure) or non-CV surgery.

- Acute coronary syndrome

ACS includes MI and unstable angina.

MI (non-fatal)

The term MI should be used when there is evidence of myocardial necrosis in a clinical setting consistent with myocardial ischemia. Under these conditions, any one of the following criteria (A to C) meets the diagnosis for myocardial infarction.

A. Spontaneous MI (type 1)

To identify a type 1 MI, patients should demonstrate spontaneous symptoms of myocardial ischemia unprovoked by supply/demand inequity, together with ≥1 of the following criteria:

• Cardiac biomarker elevation: Troponin is the preferred marker for adjudicating the presence of acute MI. At least one value should show a rise and/or fall from the lowest cut-point providing 10% imprecision (typically the upper reference limit for the troponin run per standard of clinical care). Creatine kinase-MB is a secondary choice of marker to troponin; a rise in CK-MB above the local upper reference limit would be consistent with myocardial injury.

• ECG changes consistent with new ischemic changes

– ECG changes indicative of new ischemia (new ST-T changes or new left bundle branch block [LBBB]) or ECG manifestations of acute myocardial ischemia (in the absence of left ventricular hypertrophy [LVH] and LBBB):

– Development of pathological Q waves in the ECG

1. Any Q-wave in leads V2–V3 ≥0.02 seconds or QS complex in leads V2 and V3
2. Q-wave ≥0.03 seconds and ≥0.1 mV deep or QS complex in leads I, II, aVL, aVF, or V4-V6 in any two leads of a contiguous lead grouping (I, aVL, V6; V4-V6; II, III, and aVF)

– ST elevation: New ST elevation at the J-point in two contiguous leads with the cut-off points: ≥0.2 mV in men or ≥0.15 mV in women in leads V2–V3 and/or ≥0.1 mV in other leads

– ST depression and T-wave changes: New horizontal or down-sloping ST depression ≥0.05 mV in two contiguous leads and/or T inversion ≥0.1 mV in two contiguous leads with prominent R-wave or R/S ratio >1

• Imaging evidence of new non-viable myocardium or new wall motion abnormality

B. “Demand”-related (type 2) MI

Patients with type 2 MI should be considered under similar diagnostic criteria as a type 1 MI; however, type 2 MI should be considered present when myocardial ischemia and infarction are consequent to supply/demand inequity, rather than a spontaneous plaque rupture and coronary thrombosis.

C. Percutaneous coronary intervention (PCI)-related MI (type 4a/4b)

For PCI in patients with normal baseline troponin values, elevations of cardiac biomarkers above the 99th percentile URL within 24 hours of the procedure are indicative of peri-procedural myocardial necrosis. By convention, increases of biomarkers >3 × 99th percentile URL (troponin or CK-MB >3 × 99th percentile URL) are consistent with PCI-related MI.

Where the cardiac biomarker is elevated prior to PCI, a ≥20% increase in the value of the second cardiac biomarker sample within 24 hours of PCI and documentation that cardiac biomarker values were decreasing (two samples ≥6 hours apart) prior to the suspected recurrent MI are consistent with PCI-related MI.

Symptoms of cardiac ischemia are not required.

D. Coronary artery bypass grafting (CABG)-related MI (type 5)

For CABG in patients with normal baseline troponin values, elevation of cardiac biomarkers above the 99th percentile URL within 72 hours of the procedure is indicative of peri-procedural myocardial necrosis. By convention, an increase of biomarkers >5 × 99th percentile URL (troponin or CK-MB >5 × 99th percentile URL) plus at least one of the following is consistent with CABG-related MI:

• New pathological Q waves in at least two contiguous leads on the ECG that persist for 30 days, or new LBBB

• Angiographically documented new graft or native coronary artery occlusion

• Imaging evidence of new loss of viable myocardium

If the cardiac biomarker is elevated prior to CABG, a ≥20% increase in the value of the second cardiac biomarker sample within 72 hours of CABG and documentation that cardiac biomarker values were decreasing (two samples ≥6 hours apart) prior to the suspected recurrent MI plus new pathological Q-waves in ≥2 contiguous leads on the electrocardiogram; or new LBBB, angiographically documented new graft, or native coronary artery occlusion; or imaging evidence of new loss of viable myocardium are consistent with a periprocedural MI after CABG. Symptoms of cardiac ischemia are not required.

Clinical classification of acute MI. Every MI identified by the CEC will be classified into one of the following categories:

• Type 1: Spontaneous MI related to ischemia arising from a primary coronary event such as plaque erosion and/or rupture, fissuring, or dissection

• Type 2: MI secondary to ischemia attributable to either increased oxygen demand or decreased supply, e.g., coronary artery spasm, coronary embolism, anaemia, arrhythmias, hypertension, or hypotension

• Type 3: Sudden unexpected cardiac death, including cardiac arrest, often with symptoms suggestive of myocardial ischemia, accompanied by presumably new ST elevation, new LBBB, or evidence of fresh thrombus in a coronary artery by angiography and/or at autopsy, with death occurring before blood samples could be obtained or before the appearance of cardiac biomarkers in the blood

• Type 4a: MI associated with PCI

• Type 4b: MI associated with stent thrombosis as documented by angiography or at autopsy

• Type 5: MI associated with CABG

Hospitalization for unstable angina

The date of this event will be the day of hospitalization of the patient including any overnight stay at an emergency room or chest pain unit. Unstable angina requiring hospitalization is defined as all of the following:

• No elevation in cardiac biomarkers (cardiac biomarkers negative for myocardial necrosis) according to conventional assays or contemporary sensitive assays

• Clinical presentation: Cardiac symptoms lasting ≥10 minutes and considered to be myocardial ischemia upon final diagnosis with one of the following:

– Rest angina

– New-onset (<2 months) severe angina (Canadian Cardiovascular Society [CCS] Grading Scale, or CCS classification system, classification severity ≥III)

– Increasing angina (in intensity, duration, and/or frequency) with an increase in severity of >1 CCS class to CCS class >III

• Angina requiring an unscheduled visit to a healthcare facility and overnight admission

• At least one of the following:

– New or worsening ST or T-wave changes by ECG. ECG changes should satisfy the following criteria for acute myocardial ischemia in the absence of LVH and LBBB:

1. ST elevation: New transient (known to be <20 minutes) ST elevation at the J-point in two contiguous leads with cut-off points of ≥0.2 mV in men or ≥0.15 mV in women in leads V2–V3 and/or ≥0.1 mV in other leads
2. ST depression and T-wave changes: New horizontal or down-sloping ST depression ≥0.05 mV in two contiguous leads; and/or T inversion ≥0.1 mV in two contiguous leads with prominent R-wave or R/S ratio >1

– Evidence of ischemia on stress testing with cardiac imaging

– Evidence of ischemia on stress testing with angiographic evidence of ≥70% lesion and/or thrombus in an epicardial coronary artery or initiation/increased dosing of antianginal therapy

– Angiographic evidence of ≥70% lesion and/or thrombus in an epicardial coronary artery

- Heart failure requiring hospitalization

The date of this event will be the day of hospitalization of the patient including any overnight stay at an emergency room or chest pain unit. Heart failure requiring hospitalization is defined as an event that meets all of the following criteria:

• Requires hospitalization defined as an admission to an inpatient unit or a visit to an emergency department that results in at least a 12-hour stay (or a date change if the time of admission/discharge is not available)

• Clinical manifestations of heart failure (new or worsening), including at least one of the followings:

– Dyspnoea

– Orthopnoea

– Paroxysmal nocturnal dyspnoea

– Oedema

– Pulmonary basilar crackles

– Jugular venous distension

– Third heart sound or gallop rhythm

– Radiological evidence of worsening heart failure

• Additional/increased therapy: at least one of the followings:

– Initiation of oral diuretic, intravenous diuretic, inotrope, or vasodilator therapy

– Up-titration of oral diuretic or intravenous therapy, if already on therapy

– Initiation of mechanical or surgical intervention (mechanical circulatory support, heart transplantation, or ventricular pacing to improve cardiac function); or the use of ultrafiltration, hemofiltration, or dialysis that is specifically directed at the treatment of heart failure

Changes in a biomarker (e.g., brain natriuretic peptide) consistent with CHF will support this diagnosis.

Transient ischemic attack (TIA)

A transient episode of neurological dysfunction caused by focal brain, spinal cord, or retinal ischemia, without acute infarction.

- Stroke

The rapid onset of a new persistent neurologic deficit attributed to an obstruction in cerebral blood flow and/or cerebral haemorrhage with no apparent non-vascular cause (e.g., trauma, tumour, or infection). Available neuroimaging studies will be considered to support the clinical impression and to determine if there is a demonstrable lesion compatible with an acute stroke. Strokes will be classified as ischemic, haemorrhagic, or unknown.

Diagnosis of stroke. For the diagnosis of stroke, the following four criteria should be fulfilled:

• Rapid onset of a focal/global neurological deficit with at least one of the following:

– Change in level of consciousness

– Hemiplegia

– Hemiparesis

– Numbness or sensory loss affecting one side of the body

– Dysphasia/aphasia

– Hemianopia (loss of half of the field of vision of one or both eyes)

– Other new neurological sign(s)/symptom(s) consistent with stroke

Note: If the mode of onset is uncertain, a diagnosis of stroke may be made provided that there is no plausible non-stroke cause for the clinical presentation

• Duration of a focal/global neurological deficit ≥24 hours OR <24 hours if attributable to at least one of the following therapeutic interventions:

– Pharmacologic (i.e., thrombolytic drug administration)

– Non-pharmacologic (i.e., neurointerventional procedure such as intracranial angioplasty)

or

– Available brain imaging clearly documents a new haemorrhage or infarct

or

– The neurological deficit results in death

• No other readily identifiable non-stroke cause for the clinical presentation (e.g., brain tumour, trauma, infection, hypoglycaemia, peripheral lesion)

• Confirmation of the diagnosis by at least one of the following: *

– Neurology or neurosurgical specialist

– Brain imaging procedure (at least one of the followings):

1. CT scan
2. MRI scan
3. Cerebral vessel angiography

– Lumbar puncture (i.e., spinal fluid analysis diagnostic of intracranial haemorrhage)

If a stroke is reported but evidence of confirmation of the diagnosis by the methods outlined above is absent, the event will be discussed at a full CEC meeting. In such cases, the event may be adjudicated as a stroke on the basis of the clinical presentation alone, but full CEC consensus will be mandatory.

If the acute focal signs represent a worsening of a previous deficit, these signs must have either

• Persisted for more than one week

OR

• Persisted for more than 24 hours and accompanied by an appropriate new CT or MRI finding

Classification of stroke. Strokes are sub-classified as follows:

• Ischemic (non-haemorrhagic): A stroke caused by an arterial obstruction attributable to either a thrombotic (e.g., large vessel disease/atherosclerotic or small vessel disease/lacunar) or embolic aetiology. This category includes ischemic stroke with haemorrhagic transformation (i.e., no evidence of haemorrhage on an initial imaging study but appearance on a subsequent scan)

• Haemorrhagic: A stroke caused by a haemorrhage in the brain as documented by neuroimaging or autopsy. This category will include strokes attributable to primary intracerebral haemorrhage (intraparenchymal or intraventricular), subdural hematoma and primary subarachnoid haemorrhage

• Not assessable: The stroke type could not be determined by imaging or other means (e.g., lumbar puncture, neurosurgery, or autopsy) or no imaging was performed.

Hypoglycaemic adverse events (requiring any intervention)

Hypoglycaemic adverse events are defined as the requirement of high-sugar food, drinks, or glucose because of a very low level of blood glucose.

Representative symptoms of hypoglycaemia may include:

– Irregular heart rhythm

– Fatigue

– Pale skin

– Shakiness

– Anxiety

– Sweating

– Hunger

– Irritability

– Tingling sensation around the mouth

– Crying out during sleep

Urinary tract infection

Urinary tract infection is defined as the requirement of antibiotics because of infectious episodes in any part of the urinary system (kidneys, ureters, bladder, or urethra).
