# Supplementary material for: Effects of luseogliflozin and voglibose on high-risk lipid profiles and inflammatory markers in diabetes patients with heart failure
Source: Sci Rep. 2022 Sep 14;12:15449. doi: 10.1038/s41598-022-19371-6 (PMC9474821; doi:10.1038/s41598-022-19371-6)
Supplement: Supplementary file 2 — Supplementary Information 2. [file 41598_2022_19371_MOESM2_ESM.docx]

**Additional file**

- Figure S1. Study design
- Figure S2. Assessments during the study period
- Table S1. Time-dependent change of biomarkers in patients with ASCVD
- Table S2. Time-dependent change of biomarkers in patients with dyslipidaemia
- Table S3. Time-dependent changes in biomarkers in patients with statin therapy at baseline
- **Additional Figures**

**Figure S1. Study design**


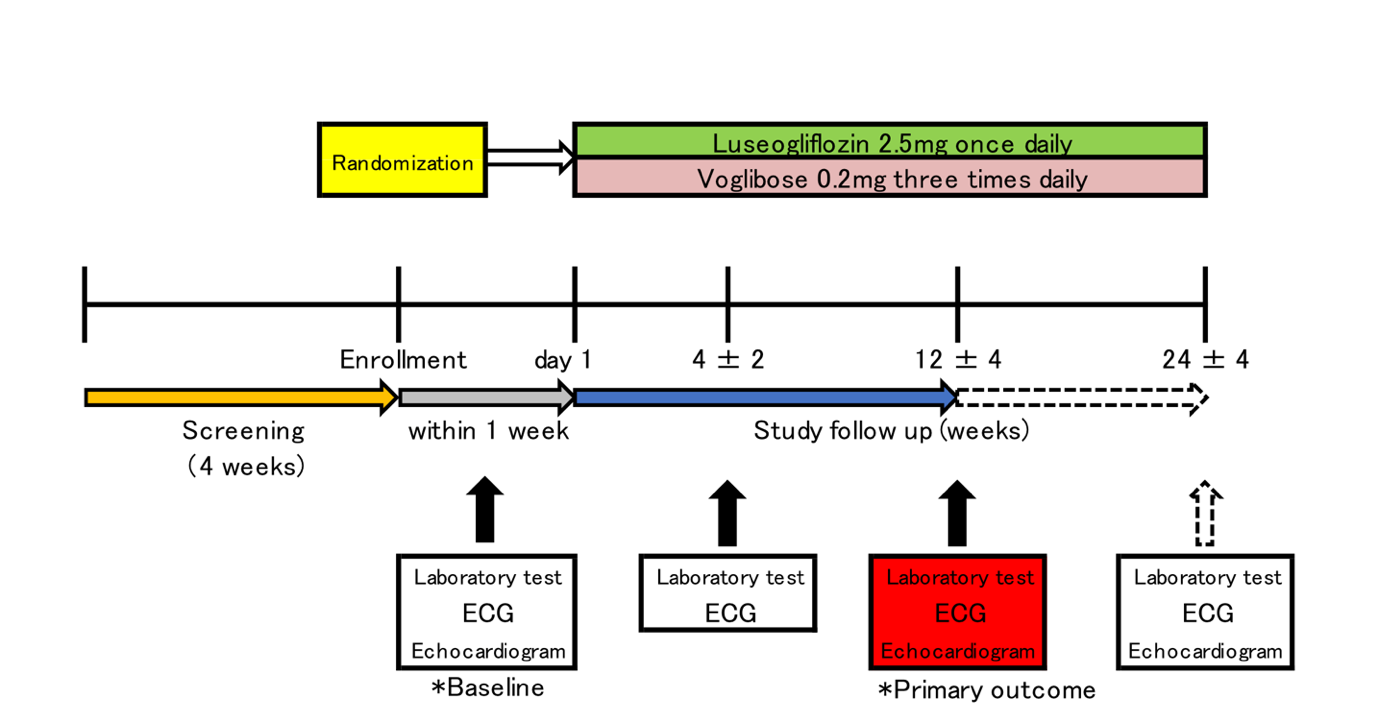


Arrows illustrate patients’ flow and the timing of follow-up. Patients with type 2 diabetes mellitus are screened whether with heart failure with preserved ejection fraction or without (screening period, yellow arrow). One of the study drugs was administered to patients met inclusion criteria after collection of baseline data within one week after randomization (grey arrow). After administration, mandatory follow-up period is for 12 weeks (study follow-up period, blue arrow). After 12 weeks, expanding follow-up are continued in patients agreed with (Arrow with dotted line).

ECG, electrocardiogram

**Figure S2. Assessments during the study period**


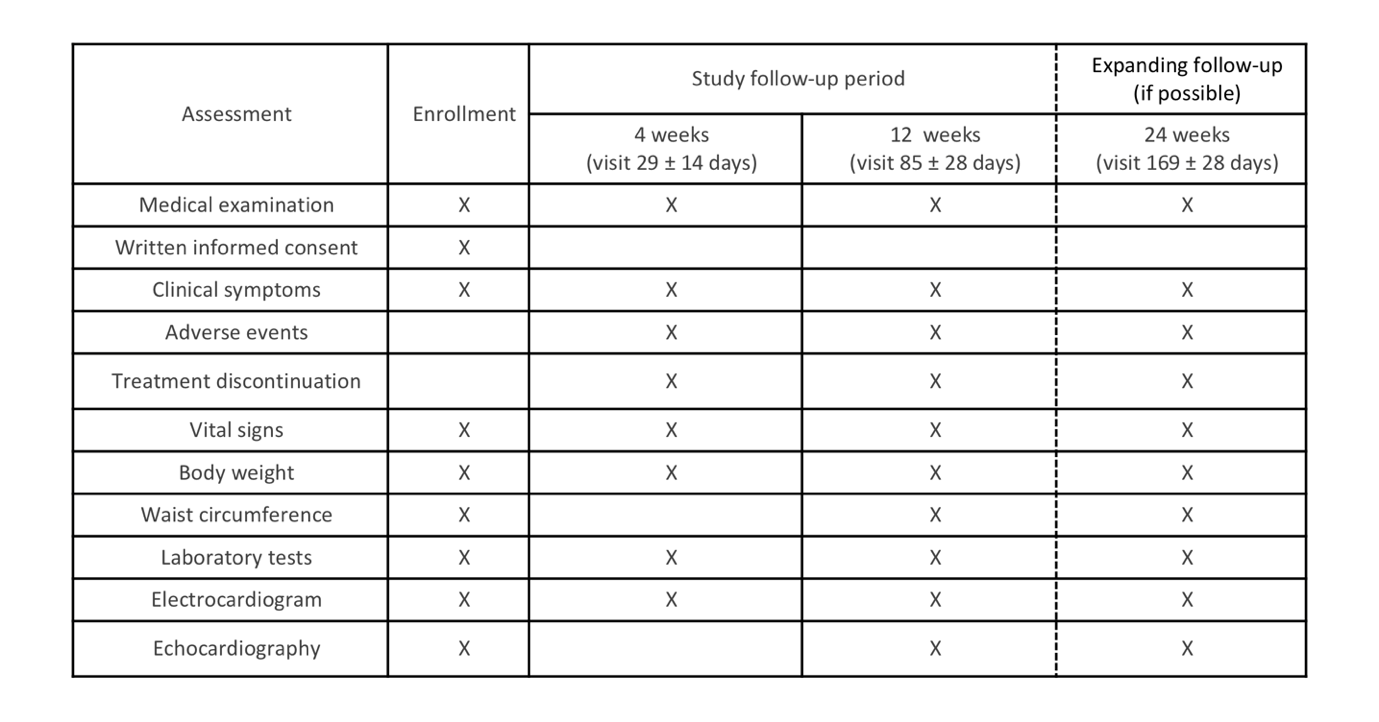


**Table S1. Time-dependent change of biomarkers in patients with ASCVD**

|  | Luseogliflozin group  (n = 44) | | | | | Voglibose group  (n = 47) | | | | |
| --- | --- | --- | --- | --- | --- | --- | --- | --- | --- | --- |
|  | Visit | | | |  | Visit | | | |  |
| Variables | Baseline | Week 4 | Week 12 | Week 24 | p value | Baseline | Week 4 | Week 12 | Week 24 | p value |
| Log MDA-LDL | 4.51 (0.36) | NA | 4.56 (0.35) | NA | 0.21 | 4.49 (5.11) | NA | 4.48 (0.32) | NA | 0.81 |
| Log small-dense LDL cholesterol | 3.36 (0.46) | NA | 3.35 (0.46) | NA | 0.94 | 3.31 (0.46) | NA | 3.25 (0.46) | NA | 0.28 |
| Log adiponectin | 2.19 (0.53) | NA | 2.18 (0.55) | NA | 0.67 | 2.36 (0.57) | NA | 2.29 (0.53) | NA | 0.06 |
| Log high-sensitivity CRP | 6.64 (1.06) | NA | 6.84 (1.15) | NA | 0.20 | 6.56 (1.52) | NA | 6.70 (1.55) | NA | 0.49 |
| Total cholesterol, mg/dl | 172.0 (31.8) | 171.8 (34.5) | 175.7 (39.6) | 172.3 (30.6) | 0.62 | 172.7 (33.1) | 163.5 (33.5) | 166.9 (32.5) | 172.7 (33.1) | 0.15 |
| HDL cholesterol, mg/dl | 51.7 (15.3) | 50.4 (18.2) | 51.7 (16.3) | 51.4 (15.4) | 0.92 | 54.1 (17.5) | 50.0 (15.1) | 51.7 (16.1) | 54.1 (17.5) | 0.68 |
| LDL cholesterol, mg/dl | 85.3 (29.9) | 89.1 (32.32) | 88.6 (33.8) | 85.9 (28.1) | 0.59 | 88.1 (26.9) | 82.6 (29.8) | 90.0 (28.8) | 88.1 (26.9) | 0.98 |
| Log triglyceride | 5.03 (0.53) | 4.95 (0.51) | 5.05 (0.53) | 5.01 (0.56) | 0.86 | 4.88 (0.57) | 4.89 (0.57) | 4.75 (0.44) | 4.77 (0.51) | 0.03 |

Data are presented as the mean (standard deviation). Log-transformed values of MDA-LDL, small-dense LDL cholesterol, adiponectin, high-sensitivity CRP, and triglyceride concentrations are shown.

^†^CRP indicates C-reactive protein; ASCVD, atherosclerotic cardiovascular disease; HDL, high-density lipoprotein; MDA-LDL, malondialdehyde low-density lipoprotein.

**Table S2. Time-dependent change of biomarkers in patients with dyslipidaemia**

|  | Luseogliflozin group  (n = 61) | | | | | Voglibose group  (n = 59) | | | | |
| --- | --- | --- | --- | --- | --- | --- | --- | --- | --- | --- |
|  | Visit | | | |  | Visit | | | |  |
| Variables | Baseline | Week 4 | Week 12 | Week 24 | p value | Baseline | Week 4 | Week 12 | Week 24 | p value |
| Log MDA-LDL | 4.50 (0.34) | NA | 4.51 (0.32) | NA | 0.75 | 4.48 (0.38) | NA | 4.47 (0.36) | NA | 0.74 |
| Log small-dense LDL cholesterol | 3.40 (0.41) | NA | 3.35 (0.42) | NA | 0.13 | 3.41 (0.46) | NA | 3.31 (0.5) | NA | 0.04 |
| Log adiponectin | 2.21 (0.49) | NA | 2.19 (0.51) | NA | 0.40 | 2.37 (0.6) | NA | 2.31 (0.57) | NA | 0.04 |
| Log high-sensitivity CRP | 6.59 (0.99) | NA | 6.88 (1.12) | NA | 0.02 | 6.51 (1.4) | NA | 6.7 (1.35) | NA | 0.24 |
| Total cholesterol, mg/dl | 173.1 (31.1) | 174.1 (31.7) | 173.8 (34.7) | 171.9 (28.9) | 0.54 | 179.3 (40.5) | 171.3 (38.7) | 170.3 (36.5) | 177.8 (41.0) | 0.37 |
| HDL cholesterol, mg/dl | 53.0 (15.2) | 52.7 (17.9) | 53.0 (16.3) | 53.8 (16.7) | 0.61 | 54.1 (16.0) | 50.0 (14.1) | 51.0 (15.4) | 54.0 (16.0) | 0.97 |
| LDL cholesterol, mg/dl | 83.4 (28.9) | 87.7 (27.9) | 85.3 (28.1) | 80.7 (27.6) | 0.38 | 93.8 (32.4) | 91.2 (35.3) | 91.4 (30.4) | 94.5 (32.7) | 0.91 |
| Log triglyceride | 5.04 (0.54) | 5.00 (0.5) | 5.02 (0.56) | 5.08 (0.53) | 0.88 | 4.91 (0.54) | 4.86 (0.57) | 4.78 (0.53) | 4.87 (0.5) | 0.19 |

Data are presented as the mean (standard deviation). Log-transformed values of MDA-LDL, small-dense LDL cholesterol, adiponectin, high-sensitivity CRP, and triglyceride concentrations are shown.

^†^CRP indicates C-reactive protein; HDL, high-density lipoprotein; MDA-LDL, malondialdehyde low-density lipoprotein.

**Table S3. Time-dependent changes in biomarkers in patients with statin therapy at baseline**

|  | Luseogliflozin group  (n = 51) | | | | | Voglibose group  (n = 53) | | | | |
| --- | --- | --- | --- | --- | --- | --- | --- | --- | --- | --- |
|  | Visit | | | |  | Visit | | | |  |
| Variables | Baseline | Week 4 | Week 12 | Week 24 | p value | Baseline | Week 4 | Week 12 | Week 24 | p value |
| Log MDA-LDL | 4.47 (0.32) | NA | 4.49 (0.33) | NA | 0.70 | 4.46 (0.37) | NA | 4.46 (0.36) | NA | 0.98 |
| Log small-dense LDL cholesterol | 3.37 (0.41) | NA | 3.31 (0.42) | NA | 0.09 | 3.37 (0.48) | NA | 3.29 (0.5) | NA | 0.13 |
| Log adiponectin | 2.18 (0.51) | NA | 2.17 (0.53) | NA | 0.72 | 2.37 (0.63) | NA | 2.30 (0.6) | NA | 0.06 |
| Log high-sensitivity CRP | 6.52 (1.06) | NA | 6.74 (1.15) | NA | 0.10 | 6.46 (1.39) | NA | 6.65 (1.45) | NA | 0.29 |
| Total cholesterol, mg/dl | 170.3 (30.3) | 169.8 (30.9) | 169.2 (32.6) | 169.0 (28.6) | 0.62 | 172.5 (34.7) | 164.3 (35.6) | 166.4 (35.8) | 173.7 (39.8) | 0.93 |
| HDL cholesterol, mg/dl | 52.1 (13.5) | 51.7 (17.1) | 53.1 (14.6) | 53.5 (15.4) | 0.19 | 53.7 (16.6) | 49.7 (15.1) | 51.2 (16.6) | 54.2 (17.3) | 0.65 |
| LDL cholesterol, mg/dl | 80.6 (26.9) | 84.9 (27.0) | 81.5 (27.1) | 80.9 (25.1) | 0.79 | 88.3 (28.3) | 84.8 (33.0) | 87.9 (30.5) | 90.7 (33.4) | 0.52 |
| Log triglyceride | 5.05 (0.56) | 4.98 (0.5) | 5.00 (0.56) | 5.03 (0.51) | 0.33 | 4.88 (0.54) | 4.85 (0.58) | 4.77 (0.53) | 4.85 (0.5) | 0.43 |

Data are presented as the mean (standard deviation). Log-transformed values of MDA-LDL, small-dense LDL cholesterol, adiponectin, high-sensitivity CRP, and triglyceride concentrations are shown.

^†^CRP indicates C-reactive protein; HDL, high-density lipoprotein; MDA-LDL, malondialdehyde low-density lipoprotein
